# A Network Analysis of the Fear of COVID-19 Scale (FCV-19S): A Large-Scale Cross-Cultural Study in Iran, Bangladesh, and Norway

**DOI:** 10.3390/ijerph19116824

**Published:** 2022-06-02

**Authors:** Oscar Lecuona, Chung-Ying Lin, Dmitri Rozgonjuk, Tone M. Norekvål, Marjolein M. Iversen, Mohammed A. Mamun, Mark D. Griffiths, Ting-I Lin, Amir H. Pakpour

**Affiliations:** 1 Faculty of Health Sciences, King Juan Carlos University, 28933 Móstoles, Spain; oscar.lecuona@urjc.es; 2 Institute of Allied Health Sciences, College of Medicine, National Cheng Kung University, Tainan 70101, Taiwan; cylin36933@gs.ncku.edu.tw; 3 Department of Public Health, College of Medicine, National Cheng Kung University, Tainan 70101, Taiwan; 4 Department of Occupational Therapy, College of Medicine, National Cheng Kung University, Tainan 70101, Taiwan; 5 Biostatistics Consulting Center, National Cheng Kung University Hospital, College of Medicine, National Cheng Kung University, Tainan 70101, Taiwan; 6 Department of Molecular Psychology, Ulm University, 89081 Ulm, Germany; dmitri.rozgonjuk@ut.ee; 7 Institute of Mathematics and Statistics, University of Tartu, 50090 Tartu, Estonia; 8 Centre on Patient-Reported Outcomes, Department of Research and Development, Haukeland University Hospital, Postboks 1400, N-5021 Bergen, Norway; tone.merete.norekval@helse-bergen.no (T.M.N.); marjolein.memelink.iversen@hvl.no (M.M.I.); 9 Department of Health and Caring Sciences, Faculty of Health and Social Sciences, Western Norway University of Applied Sciences, N-5063 Bergen, Norway; 10 CHINTA Research Bangladesh, Savar, Dhaka 1342, Bangladesh; mamunphi46@gmail.com; 11 Department of Public Health, University of South Asia, Dhaka 1212, Bangladesh; 12 Department of Public Health, Daffodil International University, Dhaka 1207, Bangladesh; 13 Department of Public Health and Informatics, Jahangirnagar University, Savar, Dhaka 1342, Bangladesh; 14 Psychology Department, Nottingham Trent University, Nottingham NG1 4FQ, UK; mark.griffiths@ntu.ac.uk; 15 Department of Pediatrics, E-Da Hospital, Kaohsiung 82445, Taiwan; 16 Social Determinants of Health Research Center, Research Institute for Prevention of Non-Communicable, Qazvin University of Medical Sciences, Qazvin 3419759811, Iran; 17 Department of Nursing, School of Health and Welfare, Jönköping University, 551 11 Jönköping, Sweden

**Keywords:** Bangladesh, COVID-19, fear, Iran, network analysis, Norway

## Abstract

The rapid spread of the coronavirus disease 2019 (COVID-19) has led to high levels of fear worldwide. Given that fear is an important factor in causing psychological distress and facilitating preventive behaviors, assessing the fear of COVID-19 is important. The seven-item Fear of COVID-19 Scale (FCV-19S) is a widely used psychometric instrument to assess this fear. However, the factor structure of the FCV-19S remains unclear according to the current evidence. Therefore, the present study used a network analysis to provide further empirical evidence for the factor structure of FCV-19S. A total of 24,429 participants from Iran (*n* = 10,843), Bangladesh (*n* = 9906), and Norway (*n* = 3680) completed the FCV-19S in their local language. A network analysis (via regularized partial correlation networks) was applied to investigate the seven FCV-19S items. Moreover, relationships between the FCV-19S items were compared across gender (males vs. females), age groups (18–30 years, 31–50 years, and >50 years), and countries (Iran, Bangladesh, and Norway). A two-factor structure pattern was observed (three items concerning physical factors, including clammy hands, insomnia, and heart palpitations; four items concerning psychosocial factors, including being afraid, uncomfortable, afraid of dying, and anxious about COVID-19 news). Moreover, this pattern was found to be the same among men and women, across age groups and countries. The network analysis used in the present study verified the two-factor structure for the FCV-19S. Future studies may consider using the two-factor structure of FCV-19S to assess the fear of COVID-19 during the COVID-19 era.

## 1. Introduction

The world has been hugely impacted by the rapid spread of coronavirus disease 2019 (COVID-19). It has infected more than 270 million people worldwide, causing over 6.3 million deaths at the time of writing [1]. Consequently, the COVID-19 pandemic has been viewed as one of the most unprecedented health crises [2], and one of the worst global crises since the Second World War [3]. Among other factors, governments and institutions have asserted age as a major risk factor [4]. Even before the World Health Organization (WHO) declared COVID-19 as a worldwide pandemic, some countries implemented early responses to inhibit the spread of the virus (e.g., border controls, quarantines or lockdowns, school closures, social distancing, and remote working) [5,6]. These early responses implemented by governmental authorities markedly (and suddenly) changed people’s everyday lives. Unfortunately, such implementations are likely to increase psychological distress because they restrict gatherings and social interactions, which are among individuals’ most basic needs [7,8,9,10,11]. Therefore, vaccination appears to be the only solution to control the situation for life to go back to normal [7,12,13]. However, because of vaccine hesitancy and equality, the efficiency of COVID-19 vaccinations may not reach full potential in controlling the spread of COVID-19 [14,15,16,17]. Consequently, COVID-19 remains a serious issue for individuals. Moreover, fear of COVID-19 continues to be a public health and mental health problem [18,19,20,21].

COVID-19 (as compared with other infectious diseases) features a high rate of transmission, a high speed of mutating into different variants, and relatively high mortality due to its epidemic nature [22,23,24,25]. With such features, fear of COVID-19 has been reported across countries [26,27,28]. Regarding psychological mechanisms, fear of COVID-19 can be caused by the disease’s (i) fast and invisible transmission (i.e., individuals do not know whether they are in an environment with a high risk of infection); and (ii) complications and mortality (i.e., individuals may fear the serious consequences resulting from COVID-19). Indeed, news and media information regarding COVID-19 appear to be associated with fear of COVID-19, given that most news and media information provide anxiety-inducing stories, such as the high transmission and mortality rates [19,29,30,31]. Consequently, other psychosocial challenges and behaviors are likely to be accompanied by an increase in fear of COVID-19, including stigma, discrimination, and loss [7,8,9,10,11,32]. If fear of COVID-19 can be controlled at a low level, the protection motivation theory suggests that such fear may lead to good practices, such as preventative behaviors (e.g., wearing a mask, social distancing, washing hands, etc.) [33]. However, if fear of COVID-19 cannot be controlled, and it remains high, this may lead to socially disruptive behaviors, such as potential increases in social conflicts [34], prison riots [35], food shortages [32,36], or dissemination of COVID-19-related conspiracy theories [37], which can lead to distrust in science [38,39]. In this regard, assessing the fear of COVID-19 is an important topic in the current COVID-19 pandemic era.

Ahorsu et al. [40] identified the importance of the fear of COVID-19. They developed and validated the seven-item Fear of COVID-19 Scale (FCV-19S) to assess the fear of COVID-19. Currently, there are over 20 language versions of the FCV-19S and much evidence has been reported regarding its psychometric properties [26,41,42]. More specifically, this includes primary psychometric testing methods that assess the validity of FCV-19S, such as exploratory factor analyses (EFA), confirmatory factor analyses (CFA) [42], Rasch analysis, and the item response theory analysis [26,43]. Much of the current evidence regarding the factor structure of the FCV-19S suggests a one-factor solution but some studies have outlined error variances in the one-factor structure [44,45,46,47,48]. Some evidence instead suggests a two-factor solution, including a psychosocial (or psychological) factor (Items 1, 2, 4, 5) and a physical (or physiological) factor (Items 3, 6, 7) [49,50,51,52,53]. Other evidence suggests a bifactor model (i.e., having a general factor of fear and two specific factors of physical and psychosocial fear) [41,54,55,56]. In brief, the FCV-19S has robust psychometric properties, but the relationships among the seven items remain unclear. Therefore, using an alternative to explore the FCV-19S regarding its seven items is of great psychometric interest.

A novel way to further explore the FCV-19S is to utilize a psychometric network analysis because it has the features of “structure, positions, and dyadic properties and the overall ‘shape’ of ties on graph-theoretic properties” [57] (p. 894). Network analyses—importing frameworks in social sciences and graph theory—are applied to psychology research mainly as correlations between variables (also known as psychometric networks [58]).

In brief, a network analysis assumes psychological variables (or ‘nodes’) to correlate (or ‘edges’) between each other. This allows one to explore and model complex sets of multivariate data, flexibly and robustly, without needing strong theoretical models [58]. Some applied examples are present in psychopathology [59], personality [60], social psychology [61], and psychometric research [62,63]. Therefore, a network analysis can help researchers quickly understand the relationships between variables and provide exploratory interactive models [64]. More specifically, a network analysis can explore how the variables (e.g., the present study’s seven FCV-19S items) covary with each other. In addition, a network analysis allows researchers to map the centrality of each item (i.e., the relative importance) and the potential existence of groups of items representing latent variables or just clusters of items. Finally, networks can be estimated and compared for subsamples (e.g., age groups, gender, country of residence), allowing researchers to explore variations (or lack thereof) in measurement. These properties appear useful to further explore the psychometric properties of the FCV-19S. With these promising features, the present study examined the associations of the seven fear of COVID-19 items utilizing a psychometric network approach [65,66].

Consequently, the present study was conducted to explore and understand the specific item features in the FCV-19S utilizing a network analysis. Moreover, the present study compared the FCV-19S networks by gender (males vs. females), country (Iran, Bangladesh, and Norway), and age group (18–30 years, 31–50 years, and over 50 years). Therefore, a deeper and more complex study of the FCV-19S is provided, improving previous literature investigating its psychometric properties.

## 2. Materials and Methods

### 2.1. Participants and Study Procedure

**Figure 1:**
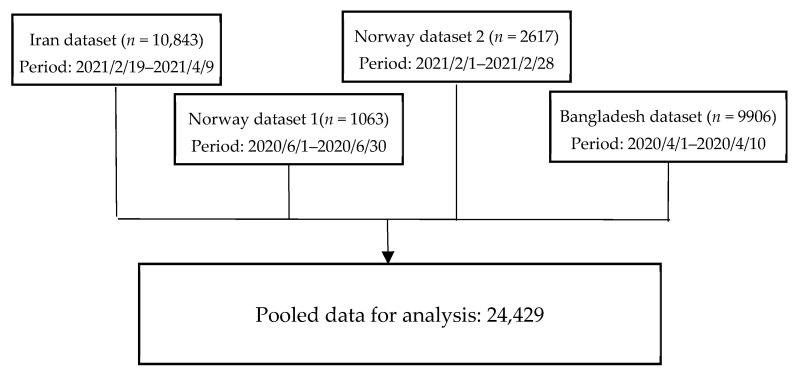
Data collection in four datasets.

The present study pooled data (*n* = 24,429) from four cross-sectional datasets from Iran (one dataset), Bangladesh (one dataset), and Norway (two datasets). Each dataset is described below. The study procedure is visualized in Figure 1.

#### 2.1.1. Iran

The Iranian data included the general population of residents in Qazvin province (a city near Tehran), Iran. The data were representative of all residents in Qazvin province (*n* = 10,843). A multistage stratified cluster sampling approach was used to collect data from adults (aged 18 years or older) in Qazvin between 19 February and 9 April 2021. The data collection procedure was approved by the Ethics Committee of Qazvin University of Medical Sciences (IR.QUMS.REC.1399.418) and all study participants provided written informed consent before enrollment [67].

#### 2.1.2. Bangladesh

The Bangladeshi data were collected nationwide through a web-based platform using social media among 64 districts of Bangladesh between 1 and 10 April 2020. All residents in Bangladesh (aged 10 years and above) were eligible to participate in the study. A total of 10,067 participants completed the study measure. However, minors (i.e., <18 years old, *n* = 152) were excluded from the present study to align the ages, i.e., to be similar to participants from Iran and Norway. A total of 9907 Bangladeshi participants’ data were used for the present data analysis. The Ethical Committee of Jahangirnagar University, Bangladesh (BBEC, JU/M 2O20/COVlD-l9/(9)2) and the Institute of Allergy and Clinical Immunology of Bangladesh ethics board, Bangladesh (IRBIACIB/CEC/03202005), approved the data collection procedure. All participants provided their written informed consent before study initiation.

#### 2.1.3. Norway

The Norwegian data was composed of two samples. The first sample was a representative sample of adult inhabitants in the city of Bergen in western Norway who were invited in April 2020 to participate in a study surveying the effects of the lockdown during the COVID-19 pandemic [68]. For the present study, 1500 individuals were randomly selected to participate in a follow-up survey in June 2020. A total of 1089 (73%) participated in the second wave. As 26 participants either did not respond to any items in this follow-up survey or did not respond to the FCV-19S, 1063 individuals were available for analysis. The study was approved by the Norwegian Regional Committee for Ethics in Medical Research (REK 2020/131560). All participants provided informed consent by responding to the emailed survey; confidentiality and the right to withdraw from the study were assured [51].

The second sample from Norway was composed of targeted undergraduate nursing students from five universities in February 2021. All full-time and part-time baccalaureate nursing students aged 18 years of age or older from five Norwegian universities at ten different campuses (N = 2617) were invited to take part in a web-based cross-sectional survey. The students consented to participate by responding to the survey. The respondents’ IP addresses were not registered, and their answers could not be linked to their identities in any way. Therefore, their participation was anonymous and ethical approval was not required according to Norwegian legislation [69].

### 2.2. Measure: Fear of COVID-19 Scale (FCV-19S)

The FCV-19S contains seven items rated on a five-point Likert scale. A higher score in the FCV-19S indicates a greater level of fear of COVID-19 [40]. The original FCV-19S was developed in Persian, and then it was translated into Bangla and Norwegian with promising psychometric properties and measurement invariance across countries [51,70]. Therefore, the FCV-19S used in the present study was appropriate for collecting information from Iranian (Persian-speaking), Bangladeshi (Bangla-speaking), and Norwegian (Norwegian-speaking) populations.

### 2.3. Data Analyses

Descriptive statistics were obtained for all variables and plots were examined (for the FCV-19S items, central tendency, and dispersion statistics are provided). To explore the relationships between items, regularized partial correlation networks (RPCNs [71]) were implemented. RPCNs import the network analysis framework to relationships between psychological variables. Therefore, network nodes (the basic components of the network) represent variables, and edges (links between nodes) represent partial regularized correlations. Mathematically, RPCNs take a correlation matrix (in this case, the polychoric correlation matrix since all items were ordinal) as an input to partialize all correlations for all other present correlations in the matrix. It then regularizes the false positive rate of the network forcing near-zero correlations to zero, assuming them to be essentially uncorrelated. The standard estimation method is the expected Bayesian inference criteria (EBIC) with the graphical least absolute shrinkage optimization (gLASSO) regularization. However, power and replicability analyses indicated problems of specificity in the present sample, so an alternative estimation method was chosen that showed high specificity and sensitivity, namely, the unregularized Gaussian graphical model search and selection (‘GGMModSelect’; see Supplementary Materials for details) [72]. This method starts with a network estimated by the gLASSO, and iteratively adds and removes edges until the EBIC can no longer be improved. All subsequent analyses were performed with the estimated network with ‘GGMModSelect’. In addition, a proportion of explained variances for each node by the network can be obtained with the R^2^ statistic. The final product of this process is a partial regularized correlation matrix, named the ‘weight matrix’.

**Figure 2:**
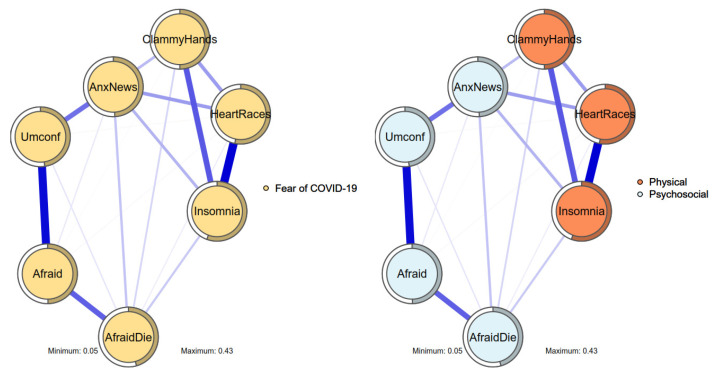
Estimated network of the FFMQ with the 1- and 2-factor solution Fruchterman–Reingold method. Note: Items are rounded in circles, with pies representing the explained variance (R^2^) of each item. Lines connecting items represent correlations, blue = positive correlations, with thicker lines representing stronger correlations. Highly correlated items tend to be closer, while non-correlated nodes tend to be farther.

Following Mullarkey et al. [73], item standard deviations (with an exclusion criterion below 2.5 SDs of standard deviations) were examined since small-variance items can influence the final estimates of the network. Moreover, conceptually or empirically overlapping items can bias network estimations since they could measure latent traits without relevant, unique content. Therefore, empirically overlapping items with the three-step approach were searched for. More specifically, the research team (i) selected pairs of items with potential conceptual overlap and estimated their correlations, (ii) obtained their mean and SD to form a criterion of 1 SD as empirically overlapping items, and (iii) created composite scores for each resulting pair, reducing the network. No items met the criteria. To assess network replicability in-depth, bootstrap techniques to the network were applied. This allowed obtaining confidence intervals for edges and other relevant statistics (provided in the results when available). In addition, the correlation stability (CS) coefficient was implemented to assess the stability of edges, with values above 0.25 as a minimum, and values above 0.5 as an ideal [71]. Once the replicability of the network was assessed, the network was graphically represented (see Figure 2 for an example). Each node (i.e., variable) is represented with a circle with a surrounding pie representing the total explained variance by other nodes (i.e., the R^2^ statistic). Each edge (i.e., correlation) is represented with a line connecting two nodes, where color represents its sign (e.g., blue for positive and red for negative correlations) and thickness represents its magnitude (i.e., thicker lines as higher in absolute value, and thinner as lower in absolute value). To allocate the nodes, the Fruchterman–Reingold algorithm provides a graphical allocation of nodes according to their relevance in the network (i.e., the more intense correlations with each variable). This algorithm was chosen because it provides visually clear and intuitive displays (alternative methods of network plots were examined and are provided in the Supplementary Materials).

Once the network is estimated, it is relevant to assess possible groups of nodes and the relative importance of each node. To explore groups of nodes (e.g., due to latent variables), an exploratory graph analysis (EGA; [74] was implemented. This technique has shown comparable performances to traditional latent variable extraction methods, such as parallel analysis [75]. Other algorithms were also implemented to compare with the EGA default (i.e., *walktrap*)*,* such as *spinglass* and multilevel. To assess the stability of EGA, all EGAs were bootstrapped. Entropy indices [76] and CFA-based fit indices (i.e., CFI and RMSEA) were estimated and interpreted to assess model fit. Finally, an exploratory factor analysis was performed for this sample as a comparison to RPCNs and EGA estimates with a more standard technique (see Supplementary Materials for details).

To explore the relative importance of each node, a centrality analysis was implemented. Centrality involves the intensity, closeness, and inter-connectivity or betweenness of each node. Due to bias and interpretation issues when latent variables are involved [77], standard centrality indices were not considered for structures with more than one cluster or factor. Instead, bridge centrality indices were implemented [78]. These centrality indices allow assessing to which degree each node is relatively important within the network without inflation due to common factors, such as item clusters. To do so, they extract the variance explained in each node due to variables from the same cluster, allowing for an assessment of relative importance without inflation due to latent variables. Therefore, the centrality of each node can be estimated without the inflation of relative importance due to clusters. In addition, the degree to which items from different clusters interact with each other can be assessed.

To compare networks by gender, age group, and country, subset networks between males and females, between young and old, and between Iran, Bangladesh, and Norway were estimated. To do this, the joint graphical lasso analysis, using the fused graphical lasso estimation method was implemented [58,79]. This method allowed to estimate a group of networks using regularization of the differences in parameters across groups (an empirical example is available in Fried [80]). All subset networks were examined in power, predictability, and dimensionality with the same procedure as the overall network. In addition, all networks were bootstrapped to enable comparison. Other analytic frameworks were discarded to do this (e.g., network comparison test) due to having large sample sizes, making *p*-values too sensitive to non-meaningful deviations. To make the visual comparison between networks intuitive, all subset networks that showed relevant differences were plotted with the average placement for each node. This way, the only visual differences were in the correlations between subsamples.

All analyses were computed using the R environment [81]. Descriptive statistics were computed using *psych* [82], while the network analyses were computed using *bootnet* [71], *mgm* [83], and *networktools* [84]. The EGAs were computed using *EGAnet* [85,86], and the EFAs were computed using *psych* [82]. The joint graphical lasso was estimated using *EstimateGroupNetwork* [79].

## 3. Results

For all samples, item descriptive statistics did not show relevant skewness except for age (Table 1). Therefore, the mean and standard deviation (SD), as their central tendency and dispersion estimators, were selected. All items had means around 2.5 and 3 (the central category) and SDs around 1. No items showed informativeness issues (none had SDs relevantly below the rest).

The FCV-19S items showed a positively correlated network (Figure 2) with moderate-to-high predictability (mean of R^2^ = 0.481, SD = 0.028). The bootstrap analysis showed a generally stable network (see Supplementary Materials), with little differences between samples, stability in case-drops, and correlation stability coefficients of 0.75, which are interpreted as stable. In addition, multi-dimensional scaling plots showed few differences with the Fruchterman–Reingold network, allowing Figure 2 to be used as an interpretable spatial network. The EGAs produced mixed results (see Supplementary Materials, for details). The default algorithm (i.e., *walktrap*) showed strong support for the one-factor structure. In addition, bootstrap analyses and dimension and stability analyses showed strong support for this structure. However, the alternative algorithms (i.e., *spinglass* and *multilevel*) showed strong support for a two-factor structure. In addition, the entropy and CFA-fit indices showed support for the two-factor structure. Finally, the EFA provided mixed support for the network. More specifically, while parallel analysis highly suggested a one-factor model, fit indices favored the two-factor model over the one-factor and three-factor models. Factor loadings also showed mixed results. All things considered, the network of the overall sample was interpreted as stable and provided an unclear structure between one and two factors. The one-factor structure provided a single ‘Fear of COVID-19’ cluster, while the two-factor structure provided a ‘Physical’ cluster, with items regarding physiological symptoms of fear (insomnia, heart palpitations, and clammy hands), and a ‘Psychosocial’ cluster, with items regarding psychological aspects (i.e., being afraid and uncomfortable with COVID-19, and being afraid to die), and more social aspects (i.e., being anxious due to COVID-19-related news) of fear.

Regarding the relative importance of nodes, centrality was estimated for the one-factor structure. While the “heart racing” and “insomnia” displayed the highest strength and expected influence, closeness and betweenness of these two items were medium or low. This was interpreted as showing intense but scarce edges (e.g., between themselves and with the “clammy hands” item). However, the items regarding anxiety—due to COVID-19 news and regarding being uncomfortable—displayed the highest closeness and betweenness with medium strength and expected influence. This was interpreted as the most connected items but with medium or light connections with the remaining items.

**Figure 3:**
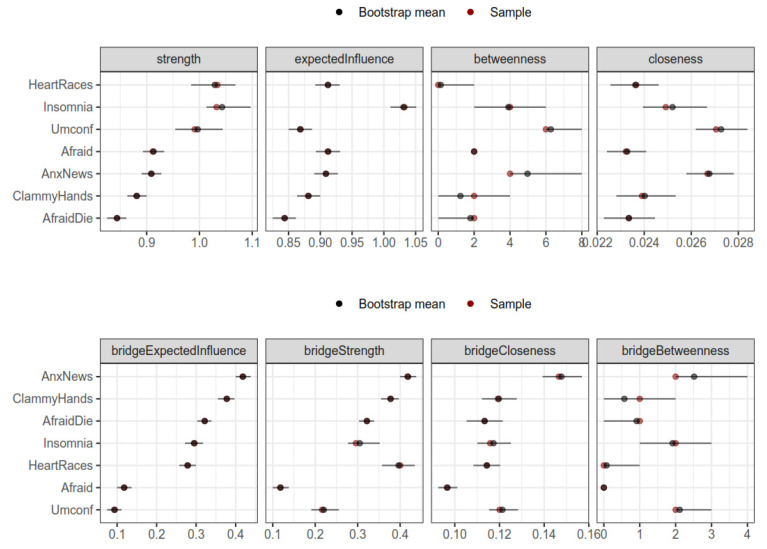
Centrality and bridge centrality indices for all items. Note: Horizontal axis represents scores in each bridge centrality index. Shaded regions represent 95% confidence intervals for the measures.

In addition, bridge centrality indices with bootstrapped confidence intervals were provided for the two-factor structure (Figure 3). The item regarding anxiety due to COVID-19-related news was the most central of all metrics. This is interpreted as the item showing several relevant connections with the ‘Physical’ cluster (i.e., insomnia, heart palpitations, and clammy hands). The second most central item regarded clammy hands, with a high bridge expected to influence and strength, but lower values in bridge centrality and betweenness. This was interpreted as this item showing relevant but scarce connections with the ‘Psychosocial’ cluster (more concretely, with being afraid to die, and being anxious due to COVID-19 news). These results are convergent with the centrality indices, indicating that possibly the most central (i.e., relevant) item of the network is the one regarding anxiety due to COVID-19 news.

### Comparison of Networks

**Figure 4:**
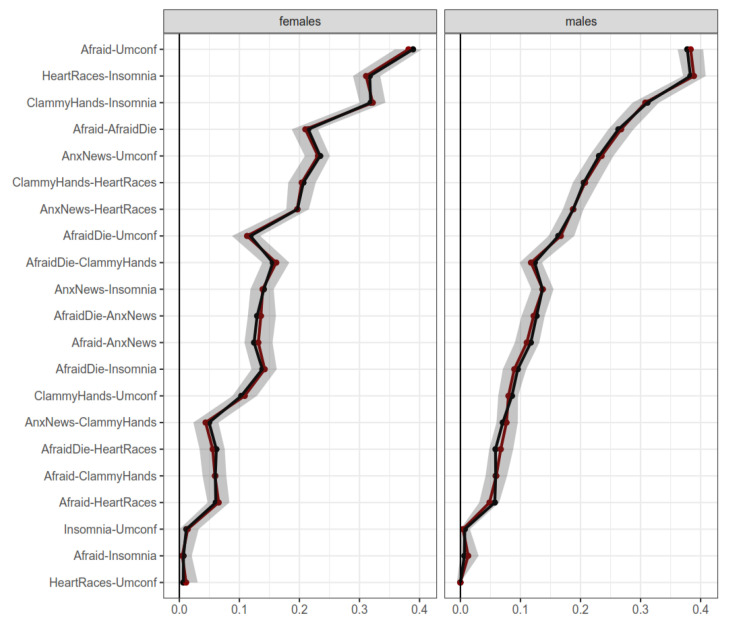
Bootstrapped edges for networks of males and females. Note: Horizontal axis represents edge weight. Shaded regions represent 95% confidence intervals for the measures.

Power and replicability analysis obtained similar levels to the overall network in all samples. More specifically, really high levels of specificity, sensitivity, and correlation between samples, along with high levels of replicated parameters (see Supplementary Materials, for details). Therefore, all subset samples appeared to be stable and replicable. Regarding gender, males and females showed little or no differences in most edges. Figure 4 displays bootstrapped edges in both samples. Significant differences **in** correlations (this is, non-overlapping confidence intervals) between males and females were mostly due to large sample sizes, which enabled narrow confidence intervals, but with small effect sizes. In addition, both samples found strong support for a one-cluster solution (see Supplementary Materials). Therefore, it can be interpreted that the FCV-19S seems to be mostly invariant regarding gender in its structure, with little or negligible differences.

**Figure 5:**
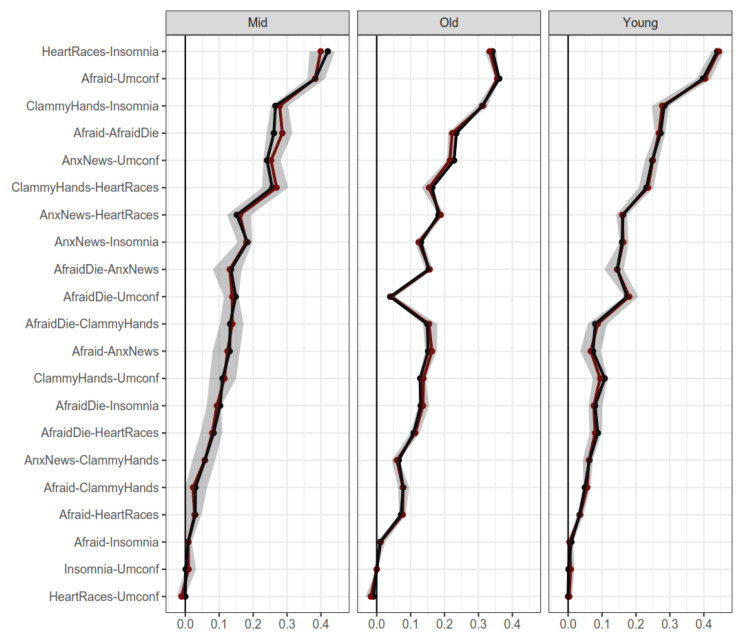
Bootstrapped edges for networks of the young age (18–29 years old), middle age (30–49 years old), and old age (>50 years old). Note: Horizontal axis represent edge weight. Shaded regions represent 95% confidence intervals for the measures.

Regarding age groups, Figure 5 displays bootstrapped edges for young- (18–29 years), mid- (30–49 years), and old- (50+ years) age groups. The edges display mostly the same values with slight differences (although more pronounced than between males and females). In addition, dimensionality was strongly supported as a two-cluster solution in all age groups. The dimensionality of structures in these samples was also stable (see Supplementary Materials). Therefore, it can be interpreted that the FCV-19S is mostly invariant regarding age groups, with little differences between age groups.

**Figure 6:**
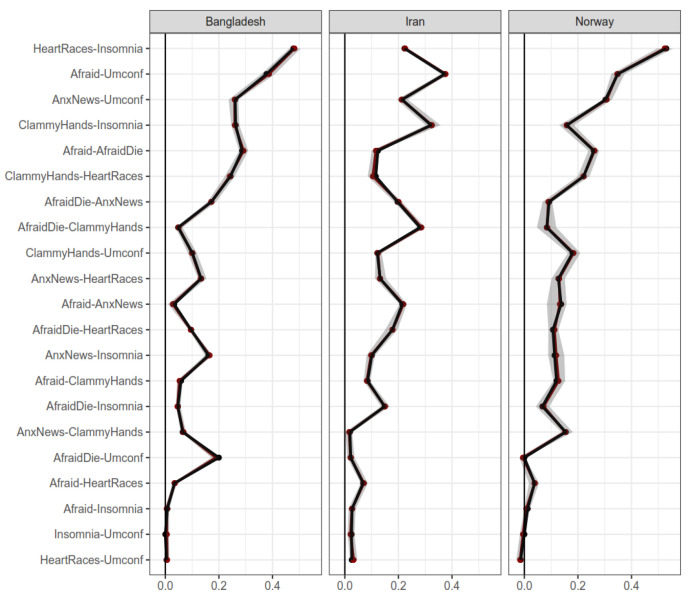
Bootstrapped edges for networks of Iran, Bangladesh, and Norway. Note: Horizontal axis represents edge weight. Shaded regions represent 95% confidence intervals for the measures.

**Figure 7:**
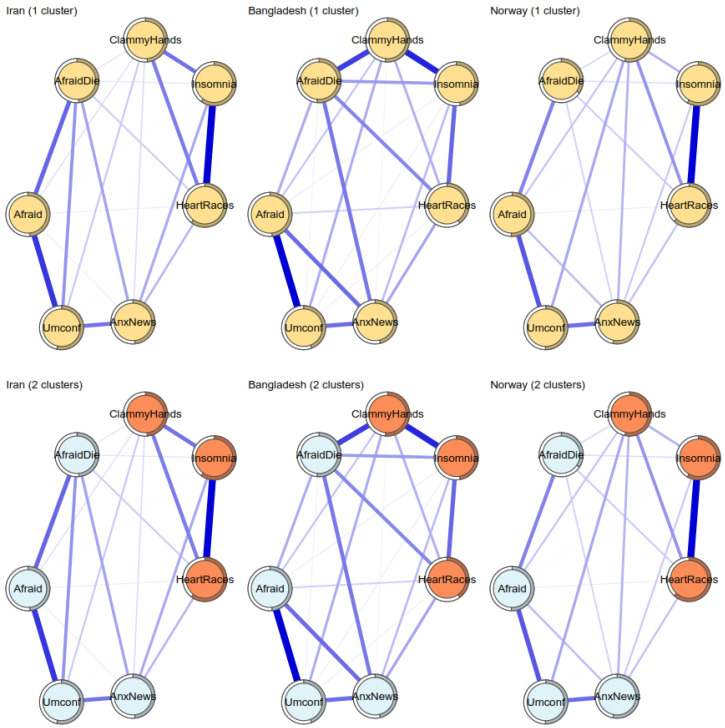
Network plots for Iran, Bangladesh, and Norway, the Fruchterman–Reingold algorithm, with an average display between groups. Note: Items are rounded in circles, with pies representing the explained variance (R^2^) of each item. Lines connecting items represent correlations, blue = positive correlations, with thicker lines representing stronger correlations. Highly correlated items tend to be closer, while non-correlated nodes tend to be farther. AfD = afraid to die; Afr = afraid; Umc = uncomfortable; AnN = anxiety from the news; ClH = clammy hands, HrR = heart racing; Ins = insomnia.

Regarding country, Figure 6 displays bootstrapped edges for the Iran, Bangladesh, and Norway samples (sorted alphabetically). Although a stable dimensionality was found in these subsamples, they showed more pronounced differences between edges than gender or age groups. These suggest that national differences may be relevant regarding fear of COVID-19 assessments. More specifically, the three subsamples seem to covary less in their edge levels. Figure 7 displays a detailed examination of network plots.

At first glance, the Norway sample seems to be less connected than the other samples. In contrast, the Bangladesh sample seems to display more intense associations between items, while the Iran sample displayed more intermediate values. The Bangladesh sample also displayed the most intense edges between clusters. However, it is important to note that all edges were positive. Finally, the dimensionality of these networks provided mixed results, not showing a clear preference for the one- or two-factor structures. Therefore, it can be interpreted that the FCV-19S may not be invariant regarding countries (regarding dimensionality and the intensity of their edges).

## 4. Discussion

To the best of the present authors’ knowledge, this is the first study to use network analyses to explore the in-depth relationships between the seven FCV-19S items across large international samples. The present study involved participants from three countries—Iran, Bangladesh, and Norway. Moreover, comparisons of the FCV-19S items among gender, age group, and country were performed. The results indicated that the FCV-19S is a stable instrument with a two-factor structure and echoed the EFA findings in the present study. The two factors correspond to prior research also indicating a two-factor structure for FCV-19S [41,54,55,56], more specifically, physical and psychosocial clusters. Moreover, the two-factor structure was found to be stable across different samples in the present study, i.e., both gender samples (male and female), all the age group samples (18–30 years, 30–50 years, and over 50 years), and all the country samples (Iran, Bangladesh, and Norway) share the two-factor structure for the FCV-19S. Given that the data collections were in different time points (e.g., Iran and one set of Norway data were collected in 2021; Bangladesh and another set of Norway data were collected in 2020) and the method used in data collection (e.g., email survey, in-person survey), the entire present data were heterogeneous in addition to the national heterogeneity. Indeed, the present data were collected with a variety of epidemiological data (e.g., different mortality rates; different confirmed cases; different vaccine coverage across countries and time periods). Such epidemiological data may impact an individual’s fear of COVID-19. However, even under such heterogeneity, the present finding showed a stable correlation structure. However, the factor structure remains as additional evidence to discard these heterogeneities as influential factors for it.

The factor structure of the FCV-19S remains unclear as the current literature provides evidence for unidimensional [44,45,46,47,48], two-factor [49,50,51,52,53], and bifactor [41,54,55,56] structures. Moreover, some arguments regarding the factor structure of the FCV-19S have been made [87] because of the different perspectives in using factor analyses to investigate the factor structure of FCV-19S. Given that the current evidence in the factor structure of FCV-19S primarily relies on factor analysis [42], using another statistical approach to further examine the factor structure of FCV-19S provides useful supplementary evidence. Consequently, the network analysis used in the present study also provides mixed evidence between the one- and two-factor structures for the FCV-19S. However, previous literature might support the two-factor structure, as concurs with the findings by Barrios et al. [49], Bitan et al. [50], Iversen et al. [51], Midorikawa et al. [52], and Reznik et al. [53].

In addition to the two-factor structure, the results from the network analysis partially support the bifactor structure of FCV-19S reported in the literature [41,54,55,56]. More specifically, the interpretable spatial network is shown in Figure 2 and Figure 4 to visualize some correlations between the physical factor items and psychosocial factor items. Therefore, all seven FCV-19S items share the same construct (i.e., fear of COVID-19), while three items have stronger linkages due to physical factors, and the remaining four items have strong linkages due to psychosocial factors. However, network analysis cannot provide further strong evidence for the bifactor structure. Moreover, the bifactor structure has been criticized that it is a better fit due to its ability to account for implausible and possibly invalid response patterns instead of accounting for the real underlying constructs [88]. Therefore, it was suggested that the FCV-19S be treated as having a two-factor structure rather than a bifactor structure. This may also simplify the interpretations of the FCV-19S. 

Identifying the FCV-19S factor structure, several implications can be made. For example, FCV-19S can help monitor the fear of COVID-19 with changing living conditions. This information can further help authorities or governments to foster tailored messages that improve public health [89]. In this example, risk perception and risk communication can be enhanced when the FCV-19S is classified as a two-factor structure. That is, the psychosocial factor of FCV-19S can be used to evaluate risk perception and risk communication, while the physical factor may let stakeholders know that the person is likely to have an irrational fear and should provide a fear reduction program for the person. Another example of using FCV-19S is that the physical factor of FCV-19S may assess parents’ irrational fear of COVID-19. With the irrational fear of COVID-19, parents are likely to protect their children in an extreme way that may subsequently jeopardize child development (e.g., social interaction reductions may cause social interaction difficulties for children) [90,91]. Therefore, FCV-19S could be a useful instrument to provide healthcare providers with important information to make good clinical decisions. Nevertheless, future studies are required to directly test additional models to obtain confirmatory evidence for the FCV-19S structure. For example, implementing current developments to integrate bifactor and EGA approaches, such as Jimenez et al. [92].

There are some limitations in the present study. First, the FCV-19S is a self-report instrument. Therefore, it cannot replace any objective measures (e.g., cortisol level and brain images) in indicating a more objective level of individual fear. Individuals may report untruthfully due to social desirability (e.g., do not want to disclose fear or report a high level of fear that calls attention to themselves). However, the use of a self-report is the most cost-effective method to roughly capture the fear in large-scale samples. Second, no other external criteria instruments (e.g., COVID stress scales [32]) were used in the present study to investigate how fear of COVID-19 associates with other relevant psychological constructs and behaviors. Therefore, evidence regarding the implications of using FCV-19S is somewhat restricted. Third, only participants from Iran, Bangladesh, and Norway were recruited. Therefore, the present study’s results cannot necessarily be generalized to the residents in other countries.

## 5. Conclusions

In conclusion, a network analysis offers evidence of a robust two-factor structure for the FCV-19S. The patterns of the physical factors (including the symptoms of clammy hands, insomnia, and heart palpitations) and the psychosocial factors (including the symptoms of being afraid, uncomfortable, afraid of dying, and anxious about COVID-19-related news) were clearly illustrated in the entire sample and the separate subsamples (including different genders, different age groups, and different countries). Therefore, future studies may consider using the two-factor structure of FCV-19S to assess fear of COVID-19 during the COVID-19 era. Potential implications related to fear can therefore be implemented. For example, when FCV-19S indicates a low level of fear, policies regarding the dissemination of accurate COVID-19 information (e.g., the negative and possibly serious consequences of COVID-19 infection) should be implemented. Then, individuals’ attitudes, attention, and awareness may possibly change and adhere to preventive behaviors. However, the fear level should be monitored carefully so as not to result in panic. Moreover, information stating the effectiveness of preventive COVID-19 behaviors in decreasing infection possibilities should be disseminated simultaneously.

## Figures and Tables

**Table 1:** Descriptive statistics of the FCV-19S.

Items	Overall (N = 24,429)		Men (N = 10,149)		Women (N = 11,657)	
	Mean	SD	Mean	SD	Mean	SD
1	3.51	1.23	3.40	1.26	3.68	1.21
2	3.53	1.19	3.35	1.23	3.67	1.16
3	2.37	1.21	2.26	1.18	2.52	1.23
4	3.04	1.39	2.93	1.36	3.37	1.34
5	3.25	1.26	3.16	1.28	3.44	1.23
6	2.29	1.21	2.00 ^a^	1.48 ^a^	2.46	1.25
7	2.51	1.30	2.49	1.28	2.69	1.33
**Items**	**Iran (N = 10,843)**		**Bangladesh (N = 9906)**		**Norway (N = 3680)**	
	**Mean**	**SD**	**Mean**	**SD**	**Mean**	**SD**
1	3.62	1.36	3.60	1.05	2.90	1.12
2	3.62	1.29	3.51	1.08	3.34	1.18
3	2.00 ^a^	1.48 ^a^	2.49	1.13	2.00 ^a^	1.48 ^a^
4	3.53	1.39	2.92	1.23	2.00 ^a^	1.48 ^a^
5	3.24	1.35	4.00 ^a^	1.08	2.56	1.17
6	2.39	1.31	2.42	1.12	1.00 ^a^	0.88
7	2.47	1.32	2.86	1.24	1.00 ^a^	0.96
**Items**	**18–30 years old** **(N = 13,494)**		**30–50 years old** **(N = 8113)**		**>50 years old** **(N = 2412)**	
	**Mean**	**SD**	**Mean**	**SD**	**Mean**	**SD**
1	3.49	1.16	3.61	1.32	3.34	1.32
2	3.55	1.13	3.59	1.26	3.30	1.27
3	2.39	1.16	2.41	1.26	2.00 ^a^	1.48 ^a^
4	2.91	1.35	3.34	1.41	2.93	1.41
5	3.33	1.19	3.26	1.34	2.92	1.33
6	2.24	1.16	2.40	1.28	2.24	1.24
7	2.57	1.30	2.51	1.32	2.31	1.28

Note. SD = standard deviation; ^a^ median and median absolute deviation (robust version) since skewness is >|1| (item 5 in the Bangladesh sample and Items 6 and 7 in the Norway sample display the SD due to median absolute deviations = 0). Item 1 = I am most afraid of COVID-19. Item 2 = It makes me uncomfortable to think about COVID-19. Item 3 = My hands become clammy when I think about COVID-19. Item 4 = I am afraid of losing my life because of COVID-19. Item 5 = When watching news and stories about COVID-19 on social media, I become nervous or anxious. Item 6 = I cannot sleep because I’m worried about getting COVID-19. Item 7 = My heart races or palpitates when I think about getting COVID-19.

## Data Availability

The data that support the findings of this study are available from the corresponding author (A.H.P.) upon reasonable request.

## Supplementary Materials

The following supporting information can be downloaded at: https://www.mdpi.com/article/10.3390/ijerph19116824/s1. References [93,94] are cited in the Supplementary Materials.

## References

[1] (2022). Worldometer COVID-19 Coronavirus Pandemic American Library Association (ALA). https://www.worldometers.info/coronavirus/.

[2] Baker R.E., Mahmud A.S., Miller I.F., Rajeev M., Rasambainarivo F., Rice B.L., Takahashi S., Tatem A.J., Wagner C.E., Wang L.F. (2022). Infectious disease in an era of global change. Nat. Rev. Microbiol..

[3] United Nations Global Cooperation Must Adapt to Meet Biggest Threat since Second World War, Secretary-General Says on International Day, as COVID-19 Transcends Borders. Secretary General. SG/SM/20058. 23 April 2020. https://www.un.org/press/en/2020/sgsm20058.doc.htm.

[4] Bonanad C., García-Blas S., Tarazona-Santabalbina F., Sanchis J., Bertomeu-González V., Fácila L., Ariza A., Núñez J., Cordero A. (2020). The effect of age on mortality in patients with COVID-19: A meta-analysis with 611,583 subjects. J. Am. Med. Dir. Assoc..

[5] Pakpour A.H., Liu C.H., Hou W.L., Chen Y.P., Li Y.P., Kuo Y.J., Lin C.Y., Scarf D. (2021). Comparing fear of COVID-19 and preventive COVID-19 infection behaviors between Iranian and Taiwanese older people: Early reaction may be a key. Front. Public Health.

[6] Shanafelt T., Ripp J., Trockel M. (2020). Understanding and addressing sources of anxiety among health care professionals during the COVID-19 pandemic. JAMA.

[7] Ahorsu D.K., Pramukti I., Strong C., Wnag H.W., Griffiths M.D., Lin C.Y., Ko N.Y. (2021). COVID-19-related variables and its association with anxiety and suicidal ideation: Differences between international and local university students in Taiwan. Psychol. Res. Behav. Manag..

[8] Hasannia E., Mohammadzadeh F., Tavakolizadeh M., Davoudian N., Bay M. (2021). Assessment of the anxiety level and trust in information resources among iranian health-care workers during the pandemic of coronavirus disease 2019. Asian J. Soc. Health Behav..

[9] Patel B.R., Khanpara B.G., Mehta P.I., Patel K.D., Marvania N.P. (2021). Evaluation of perceived social stigma and burnout, among health-care workers working in COVID-19 designated hospital of India: A cross-sectional study. Asian J. Soc. Health Behav..

[10] Pramukti I., Strong C., Sitthimongkol Y., Setiawan A., Pandin M.G.R., Yen C.F., Lin C.Y., Griffiths M.D., Ko N.Y. (2020). Anxiety and suicidal thoughts during the COVID-19 pandemic: A cross-country comparison among Indonesian, Taiwanese, and Thai university students. J. Med. Internet Res..

[11] Shirali G.A., Rahimi Z., Araban M., Mohammadi M.J., Cheraghian B. (2021). Social-distancing compliance among pedestrians in Ahvaz, South-West Iran during the Covid-19 pandemic. Asian J. Soc. Health Behav..

[12] Huang P.C., Hung C.H., Kuo Y.J., Chen Y.P., Ahorsu D.K., Yen C.F., Lin C.Y., Griffiths M.D., Pakpour A.H. (2021). Expanding Protection Motivation Theory to explain willingness of COVID-19 vaccination uptake among Taiwanese university students. Vaccines.

[13] Fan C.W., Chen J.S., Addo F.M., Adjaottor E.S., Amankwaah G.B., Yen C.F., Ahorsu D.K., Lin C.Y. (2021). Examining the validity of the Drivers of COVID-19 Vaccination Acceptance Scale using Rasch analysis. Expert Rev. Vaccines.

[14] Alimoradi Z., Lin C.Y., Pakpour A.H. (2021). Coronavirus disease-19 vaccine inequity and gross domestic product. Asian J. Soc. Health Behav..

[15] Chen I.H., Ahorsu D.K., Ko N.Y., Yen C.F., Lin C.Y., Griffiths M.D., Pakpour A.H. (2021). Adapting the Motors of Influenza Vaccination Acceptance Scale into the Motors of COVID-19 Vaccination Acceptance Scale: Psychometric evaluation among mainland Chinese university students. Vaccine.

[16] Fan C.W., Chen I.H., Ko N.Y., Yen C.F., Lin C.Y., Griffiths M.D., Pakpour A.H. (2021). Extended theory of planned behavior in explaining the intention to COVID-19 vaccination uptake among mainland Chinese university students: An online survey study. Hum. Vaccines Immunother..

[17] Yeh Y.C., Chen I.H., Ahorsu D.K., Ko N.Y., Chen K.L., Li P.C., Yen C.F., Lin C.Y., Griffiths M.D., Pakpour A.H. (2021). Measurement invariance of the Drivers of COVID-19 Vaccination Acceptance Scale: Comparison between Taiwanese and mainland Chinese-speaking populations. Vaccines.

[18] Huang C., Wang Y., Li X., Ren L., Zhao J., Hu Y., Zhang L., Fan G., Xu J., Gu X. (2020). Clinical features of patients infected with 2019 novel coronavirus in Wuhan, China. Lancet.

[19] Lin C.Y. (2020). Social reaction toward the 2019 novel coronavirus (COVID-19). Soc. Health Behav..

[20] Olashore A.A., Akanni O.O., Fela-Thomas A.L., Khutsafalo K. (2021). The psychological impact of COVID-19 on health-care workers in African Countries: A systematic review. Asian J. Soc. Health Behav..

[21] Patil S.T., Datar M.C., Shetty J.V., Naphade N.M. (2021). Psychological consequences and coping strategies of patients undergoing treatment for COVID-19 at a tertiary care hospital: A qualitative study. Asian J. Soc. Health Behav..

[22] Baud D., Qi X., Nielsen-Saines K., Musso D., Pomar L., Favre G. (2020). Real estimates of mortality following COVID-19 infection. Lancet Infect. Dis..

[23] Bertsimas D., Lukin G., Mingardi L., Nohadani O., Orfanoudaki A., Stellato B., Wiberg H., Gonzalez-Garcia S., Parra-Calderón C.L., Robinson K. (2020). Hellenic COVID-19 Study Group (2020). COVID-19 mortality risk assessment: An international multi-center study. PLoS ONE.

[24] Chen J., Gao K., Wang R., Wei G.W. (2021). Prediction and mitigation of mutation threats to COVID-19 vaccines and antibody therapies. Chem. Sci..

[25] Notari A. (2021). Temperature dependence of COVID-19 transmission. Sci. Total Environ..

[26] Lin C.Y., Hou W.L., Mamun M.A., da Silva J.A., Broche-Pérez Y., Ullah I., Masuyama A., Wakashima K., Mailliez M., Carre A. (2021). Fear of COVID-19 Scale (FCV-19S) across countries: Measurement invariance issues. Nurs. Open.

[27] Rajabimajd N., Alimoradi Z., Griffiths M.D. (2021). Impact of COVID-19-related fear and anxiety on job attributes: A systematic review. Asian J. Soc. Health Behav..

[28] Sharma R., Bansal P., Chhabra M., Bansal C., Arora M. (2021). Severe acute respiratory syndrome coronavirus-2-associated perceived stress and anxiety among indian medical students: A cross-sectional study. Asian J. Soc. Health Behav..

[29] Bhuiyan A.I., Sakib N., Pakpour A.H., Griffiths M.D., Mamun M.A. (2020). COVID-19-related suicides in Bangladesh due to lockdown and economic factors: Case study evidence from media reports. Int. J. Ment. Health Addict..

[30] Lin C.Y., Broström A., Griffiths M.D., Pakpour A.H. (2020). Investigating mediated effects of fear of COVID-19 and COVID-19 misunderstanding in the association between problematic social media use and distress/insomnia. Internet Interv..

[31] Qiu J., Shen B., Zhao M., Wang Z., Xie B., Xu Y. (2020). A nationwide survey of psychological distress among Chinese people in the COVID-19 epidemic: Implications and policy recommendations. Gen. Psychiatry.

[32] Taylor S., Landry C.A., Paluszek M.M., Fergus T.A., McKay D., Asmundson G.J. (2020). Development and initial validation of the COVID Stress Scales. J. Anxiety Disord..

[33] Rogers R.W., Prentice-Dunn S., Gochman D.S. (1997). Protection motivation theory. Handbook of Health Behavior Research 1: Personal and Social Determinants.

[34] Bloem J.R., Salemi C. (2021). COVID-19 and Conflict. World Dev..

[35] Pattavina A., Palmieri M.J. (2020). Fears of COVID-19 contagion and the Italian prison system response. Vict. Offenders.

[36] Benton T.G. (2020). COVID-19 and disruptions to food systems. Agric. Hum. Values.

[37] Imhoff R., Lamberty P. (2020). A bioweapon or a hoax? The link between distinct conspiracy beliefs about the Coronavirus disease (COVID-19) outbreak and pandemic behavior. Soc. Psychol. Pers. Sci..

[38] Dohle S., Wingen T., Schreiber M. (2020). Acceptance and adoption of protective measures during the COVID-19 pandemic: The role of trust in politics and trust in science. Soc. Psychol. Bull..

[39] Plohl N., Musil B. (2021). Modeling compliance with COVID-19 prevention guidelines: The critical role of trust in science. Psychol. Health Med..

[40] Ahorsu D.K., Lin C.Y., Imani V., Saffari M., Griffiths M.D., Pakpour A.H. (2020). The fear of COVID-19 scale: Development and initial validation. Int. J. Ment. Health Addict..

[41] Chen I.H., Chen C.Y., Zhao K.Y., Gamble J.H., Lin C.Y., Griffiths M.D., Pakpour A.H. (2022). Psychometric evaluation of Fear of COVID-19 Scale (FCV-19S) among Chinese primary and middle schoolteachers, and their students. Curr. Psychol..

[42] Sawicki A.J., Żemojtel-Piotrowska M., Balcerowska J.M., Sawicka M.J., Piotrowski J., Sedikides C., Jonason P.K., Maltby J., Adamovic M., Agada M.G. (2022). The Fear of COVID-19 Scale: Its structure and measurement invariance across 48 countries. Psychol. Assess..

[43] Ullah I., Tahir M.J., Ali S., Waseem R., Griffiths M.D., Mamun M.A., Lin C.Y., Pakpour A.H. (2021). COVID-19 fear among Pakistanis: Psychometric evaluation of the Fear of COVID-19 Scale using Item response theory and confirmatory factor analysis. Int. J. Ment. Health Addict..

[44] Alyami M., Henning M., Krägeloh C.U., Alyami H. (2021). Psychometric evaluation of the Arabic version of the Fear of COVID-19 Scale. Int. J. Ment. Health Addict..

[45] Cavalheiro F.R.S., Sticca M.G. (2020). Adaptation and validation of the Brazilian version of the fear of COVID-19 scale. Int. J. Ment. Health Addict..

[46] Chang K.C., Hou W.L., Pakpour A.H., Lin C.Y., Griffiths M.D. (2022). Psychometric testing of three COVID-19-related scales among people with mental illness. Int. J. Ment. Health Addict..

[47] Mailliez M., Griffiths M.D., Carre A. (2021). Validation of the French version of the Fear of COVID-19 Scale and its associations with depression, anxiety and differential emotions. Int. J. Ment. Health Addict..

[48] Wakashima K., Asai K., Kobayashi D., Koiwa K., Kamoshida S., Sakuraba M. (2020). The Japanese version of the Fear of COVID-19 Scale: Reliability, validity, and relation to coping behavior. PLoS ONE.

[49] Barrios I., Ríos-González C., O’Higgins M., González-Urbieta I., García O., Almirón-Santacruz J., Navarro R., Melgarejo O., Ruiz Díaz N., Castaldelli-Maia J.M. (2021). Psychometric properties of the Spanish version of the Fear of COVID-19 Scale in Paraguayan population. Ir. J. Psychol. Med..

[50] Bitan D.T., Grossman-Giron A., Bloch Y., Mayer Y., Shiffman N., Mendlovic S. (2020). Fear of COVID-19 Scale: Psychometric characteristics, reliability and validity in the Israeli population. Psychiatry Res..

[51] Iversen M.M., Norekvål T.M., Oterhals K., Fadnes L.T., Mæland S., Pakpour A.H., Breivik K. (2021). Psychometric properties of the Norwegian version of the Fear of COVID-19 Scale. Int. J. Ment. Health Addict..

[52] Midorikawa H., Aiba M., Lebowitz A., Taguchi T., Shiratori Y., Ogawa T., Takahashi A., Takahashi S., Nemoto K., Arai T. (2021). Confirming validity of the Fear of COVID-19 Scale in Japanese with a nationwide large-scale sample. PLoS ONE.

[53] Reznik A., Gritsenko V., Konstantinov V., Khamenka N., Isralowitz R. (2021). COVID-19 fear in Eastern Europe: Validation of the Fear of COVID-19 Scale. Int. J. Ment. Health Addict..

[54] Caycho-Rodríguez T., Tomás J.M., Barboza-Palomino M., Ventura-León J., Gallegos M., Reyes-Bossio M., Vilca L.W. (2021). Assessment of fear of COVID-19 in older adults: Validation of the Fear of COVID-19 Scale. Int. J. Ment. Health Addict..

[55] Huarcaya-Victoria J., Villarreal-Zegarra D., Podestà A., Luna-Cuadros M.A. (2022). Psychometric properties of a Spanish version of the Fear of COVID-19 Scale in general population of Lima, Peru. Int. J. Ment. Health Addict..

[56] Masuyama A., Shinkawa H., Kubo T. (2020). Validation and psychometric properties of the Japanese version of the Fear of COVID-19 Scale among adolescents. Int. J. Ment. Health Addict..

[57] Borgatti S.P., Mehra A., Brass D.J., Labianca G. (2009). Network analysis in the social sciences. Science.

[58] Borsboom D., Deserno M.K., Rhemtulla M., Epskamp S., Fried E.I., McNally R.J., Robinaugh D.J., Perugini M., Dalege J., Costantini G. (2021). Network analysis of multivariate data in psychological science. Nat. Rev. Methods Primers.

[59] Bringmann L.F., Albers C., Bockting C., Borsboom D., Ceulemans E., Cramer A., Epskamp E., Eronen E.I., Hamaker E., Kuppens P. (2022). Psychopathological networks: Theory, methods and practice. Behav. Res. Ther..

[60] Costantini G., Epskamp S., Borsboom D., Perugini M., Mõttus R., Waldorp L.J., Cramer A.O. (2015). State of the aRt personality research: A tutorial on network analysis of personality data in R. J. Res. Pers..

[61] Dalege J., Borsboom D., van Harreveld F., van der Maas H.L. (2017). Network analysis on attitudes: A brief tutorial. Soc. Psychol. Pers. Sci..

[62] Briganti G., Scutari M., Linkowski P. (2021). Network structures of symptoms from the Zung Depression Scale. Psychol. Rep..

[63] Lecuona O., García-Rubio C., de Rivas S., Moreno-Jiménez J.E., Meda-Lara R.M., Rodríguez-Carvajal R. (2021). A network analysis of the Five Facets Mindfulness Questionnaire (FFMQ). Mindfulness.

[64] Li L., Niu Z., Mei S., Griffiths M.D. (2021). A network analysis approach to the relationship between fear of missing out (FoMO), smartphone addiction, and social networking site use among a sample of Chinese university students. Comput. Hum. Behav..

[65] Marcus D.K., Preszler J., Zeigler-Hill V. (2018). A network of dark personality traits: What lies at the heart of darkness?. J. Res. Pers..

[66] McMally R.J. (2021). Network analysis of psychopathology: Controversies and challenges. Annu. Rev. Clin. Psychol..

[67] Ahorsu D.K., Lin C.Y., Yahaghai R., Alimoradi Z., Broström A., Griffiths M.D., Pakpour A.H. (2022). The mediational role of trust in the healthcare system in the association between generalized trust and willingness to get COVID-19 vaccination in Iran. Hum. Vaccines Immunother..

[68] Askim J., Bergström T. (2021). Between lockdown and calm down. Comparing the COVID-19 responses of Norway and Sweden. Local Gov. Stud..

[69] Beisland E.G., Gjeilo K.H., Andersen J.R., Bratås O., Bø B., Haraldstad K., Iversen M.M., Løyland B., Norekvål T.M., Riiser K. (2021). Quality of life and fear of COVID-19 in 2600 baccalaureate nursing students at five universities: A cross-sectional study. Health Qual. Life Outcomes.

[70] Sakib N., Bhuiyan A., Hossain S., Al Mamun F., Hosen I., Abdullah A.H., Sarker M.A., Mohiuddin M.S., Rayhan I., Hossain M. (2020). Psychometric validation of the Bangla Fear of COVID-19 Scale: Confirmatory factor Analysis and Rasch analysis. Int. J. Ment. Health Addict..

[71] Epskamp S., Borsboom D., Fried E.I. (2018). Estimating psychological networks and their accuracy: A tutorial paper. Behav. Res. Methods.

[72] Epskamp S., Fried E.I. (2018). A tutorial on regularized partial correlation networks. Psychol. Methods.

[73] Mullarkey M.C., Stewart R.A., Wells T.T., Shumake J., Beevers C.G. (2018). Self-dislike and sadness are central symptoms of depression in college students: A network analysis. PsyArXiv.

[74] Golino H., Epskamp S. (2017). Exploratory graph analysis: A new approach for estimating the number of dimensions in psychological research. PLoS ONE.

[75] Golino H., Shi D., Garrido L., Christensen A., Nieto M.D., Sadana R., Thiyagarajan J.A., Pérez-Molina A. (2020). Investigating the performance of exploratory graph analysis and traditional techniques to identify the number of latent factors: A simulation and tutorial. Psychol. Methods.

[76] Golino H., Moulder R., Shi D., Christensen A.P., Nieto M.D., Nesselroade J.R., Sadana R., Thiyagarajan J.A., Boker S.M. (2021). Entropy fit index: New fit measures for assessing the structure and dimensionality of multiple latent variables. Multivar. Behav. Res..

[77] Bringmann L.F., Elmer T., Epskamp S., Krause R.W., Schoch D., Wichers M., Wigman J.T.W., Snippe E. (2019). What do centrality measures measure in psychological networks?. J. Abnorm. Psychol..

[78] Jones P.J., Ma R., McNally R.J. (2021). Bridge centrality: A network approach to understanding comorbidity. Multivar. Behav. Res..

[79] Costantini G., Epskamp S., Costantini M.G. Package “EstimateGroupNetwork” (0.3.1). https://cloud.r-project.org/web/packages/EstimateGroupNetwork/EstimateGroupNetwork.pdf.

[80] Fried E.I., Eidhof M.B., Palic S., Costantini G., Huisman-van Dijk H.M., Bockting C.L.H., Engelhard I., Armour C., Nielsen A.B.S., Karstoft K.I. (2018). Replicability and Generalizability of Posttraumatic Stress Disorder (PTSD) Networks: A Cross-Cultural Multisite Study of PTSD Symptoms in Four Trauma Patient Samples. Clin. Psychol. Sci..

[81] R Development Core Team R: A Language and Environment for Statistical Computing.

[82] Rewelle W. psych: Procedures for Personality and Psychological Research.

[83] Haslbeck J.M.B., Waldorp L.J. (2015). mgm: Estimating time-varying mixed graphical models in high-dimensional data. arXiv.

[84] Jones P., Networktools: Tools for Identifying Important Nodes in Networks R Package, Version 1.2.3. https://cran-r-project.org/web/packages/networktools/.

[85] Golino H., Christensen A. (2019). EGAnet: Exploratory Graph Analysis—A Framework for Estimating the Number of Dimensions in Multivariate Data Using Network Psychometrics.

[86] Rosseel Y. (2012). lavaan: An R package for structural equation modeling. J. Stat. Softw..

[87] Pakpour A.H., Griffiths M.D., Lin C.Y. (2020). Assessing the psychological response to the COVID-19: A response to Bitan et al. “Fear of COVID-19 scale: Psychometric characteristics, reliability and validity in the Israeli population”. Psychiatry Res..

[88] Reise S.P., Kim D.S., Mansolf M., Widaman K.F. (2016). Is the bifactor model a better model or is it just better at modeling implausible responses? Application of iteratively reweighted least squares to the Rosenberg Self-Esteem Scale. Multivar. Behav. Res..

[89] Cori L., Curzio O., Adorni F., Prinelli F., Noale M., Trevisan C., Fortunato L., Giacomelli A., Bianchi F. (2021). Fear of COVID-19 for Individuals and Family Members: Indications from the National Cross-Sectional Study of the EPICOVID19 Web-Based Survey. Int. J. Environ. Res. Public Health.

[90] Lozano-Blasco R., Quilez-Robres A., Delgado-Bujedo D., Latorre-Martínez M.P. (2021). YouTube’s growth in use among children 0–5 during COVID19: The Occidental European case. Technol. Soc..

[91] Elboj-Saso C., Cortés-Pascual A., Íñiguez-Berrozpe T., Lozano-Blasco R., Quílez-Robres A. (2021). Emotional and Educational Accompaniment through Dialogic Literary Gatherings: A Volunteer Project for Families Who Suffer Digital Exclusion in the Context of COVID-19. Sustainability.

[92] Jimenez M., Abad F.J., Garcia-Garzon E., Golino H., Christensen A.P., Garrido L.E., Dimensionality assessment in generalized bi-factor structures: A network psychometrics approach PsyArXiv 2022. https://psyarxiv.com/2ujdk/.

[93] Ferrando P.J., Lorenzo-Seva U. (2017). Program FACTOR at 10: Origins, development and future directions. Psicothema.

[94] Jones P.J., Mair P., McNally R.J. (2018). Visualizing psychological networks: A tutorial in R. Front. Psychol..

