# Supplementary material for: A Network Analysis of the Fear of COVID-19 Scale (FCV-19S): A Large-Scale Cross-Cultural Study in Iran, Bangladesh, and Norway
Source: Int J Environ Res Public Health. 2022 Jun 2;19(11):6824. doi: 10.3390/ijerph19116824 (PMC9180255; doi:10.3390/ijerph19116824)
Supplement: Supplementary file 1 [file ijerph-19-06824-s001.zip › Supplementary materials.pdf]

A network analysis of the Fear of COVID-19 Scale (FC19S): Supplementary Materials

Oscar Lecuona [1] (ORCID: 0000-0003-0080-1062)

Chung-Ying Lin[2,3,4,5]

Dmitri Rozgonjuk[6,7]

Tone M Norekvål [8,9]

Marjolein M Iversen [8,9]

Mohammed A. Mamun[10,11]

Mark D. Griffiths[12]

Ting-I Lin[13,*]

Amir H Pakpour [14,15,*]

1Faculty of Health Sciences, King Juan Carlos University, Móstoles, Spain; oscar.lecuona@urjc.es

2Institute of Allied Health Sciences, College of Medicine, National Cheng Kung University, Tainan, Taiwan; cylin36933@gs.ncku.edu.tw

3Department of Public Health, College of Medicine, National Cheng Kung University, Tainan, Taiwan

4Department of Occupational Therapy, College of Medicine, National Cheng Kung University, Tainan, Taiwan

5Biostatistics Consulting Center, National Cheng Kung University Hospital, College of Medicine, National Cheng Kung University, Tainan, Taiwan

6Department of Molecular Psychology, Ulm University, Ulm, Germany; dmitri.rozgonjuk@ut.ee

7Institute of Mathematics and Statistics, University of Tartu, Tartu, Estonia

8Centre on Patient-Reported Outcomes, Department of Research and Development, Haukeland University Hospital, Postboks 1400, N-5021 Bergen, Norway; tone.merete.norekval@helse-bergen.no (TMN), marjolein.memelink.iversen@hvl.no (MMI)

9Faculty of Health and Social Sciences, Department of Health and Caring Sciences, Western Norway Uni-versity of Applied Sciences, Bergen, Norway

10Director, CHINTA Research Bangladesh, Savar, Dhaka, Bangladesh; mamunphi46@gmail.com

11Department of Public Health and Informatics, Jahangirnagar University, Savar, Dhaka, Bangladesh

12Psychology Department, Nottingham Trent University, Nottingham, UK; mark.griffiths@ntu.ac.uk

13Department of Pediatrics, E-Da Hospital, Kaohsiung, Taiwan; firecrest31@gmail.com

14Social Determinants of Health Research Center, Research Institute for Prevention of Non-Communicable , Qazvin University of Medical Sciences, Qazvin, Iran; amir.pakpour@ju.se

15Department of Nursing, School of Health and Welfare, Jönköping University, Jönköping, Sweden

*CorrespondenceDr. Ting-I Lin, Department of Pediatrics, E-Da Hospital, Kaohsiung, Taiwan. fire-crest31@gmail.com Prof.

Amir H Pakpour. E-mail: amir.pakpour@ju.se

A network analysis of the Fear of COVID-19 Scale (FC19S): Supplementary Materials

Power Analysis

We conducted a power and replicability analysis for the network. Power analysis (Figure S1) bootstrapps the network to obtain correlation between samples of the same network, sensitivity and specificity indices. In addition, replicability of centrality measures was also obtained for expected influence, betweenness, closeness and strength. Replicability of bridge centrality indices was unavailable. With the EBIC-glasso estimation method, extremely high values of correlation between samples, sensitivity and centrality values were obtained (almost 1 for our sample). However, extremely low values for specificity were also obtained (almost 0). This indicates that the EBIC-glasso seems to produce an unstable network, since false positives would be almost null, but false negatives would be total. Therefore, we tested the GGMModSelect estimation method. Power analysis indicated the same levels of correlation between samples and a slightly lower sensitivity, but higher values of specificity. Centrality indices were almost identical but with better values for strength. Thus, we conclude that the GGMModSelect estimation method produces a more stable and replicable network.

Replicability analysis (Figure S2) adds more evidence to the power analysis. It bootstraps the network to obtain indices that test the proportion of replicated non-zero edges, replicated zero edges, and a Jaccard-Tanimoto similarity coefficient (which assesses the similarity between samples). The EBIC-glasso estimation method displayed extremely high correlation between samples, replicated non-zero and zero edges, and Jaccard index. However, the GGMModSelect also provided extremely high values in all cases. Centrality displayed almost identical values in both estimation methods. Therefore, we conclude that the

GGMModSelect estimation method provides more power while similar replicability.

Subsequent analyses use the network estimated with GGMModSelect.

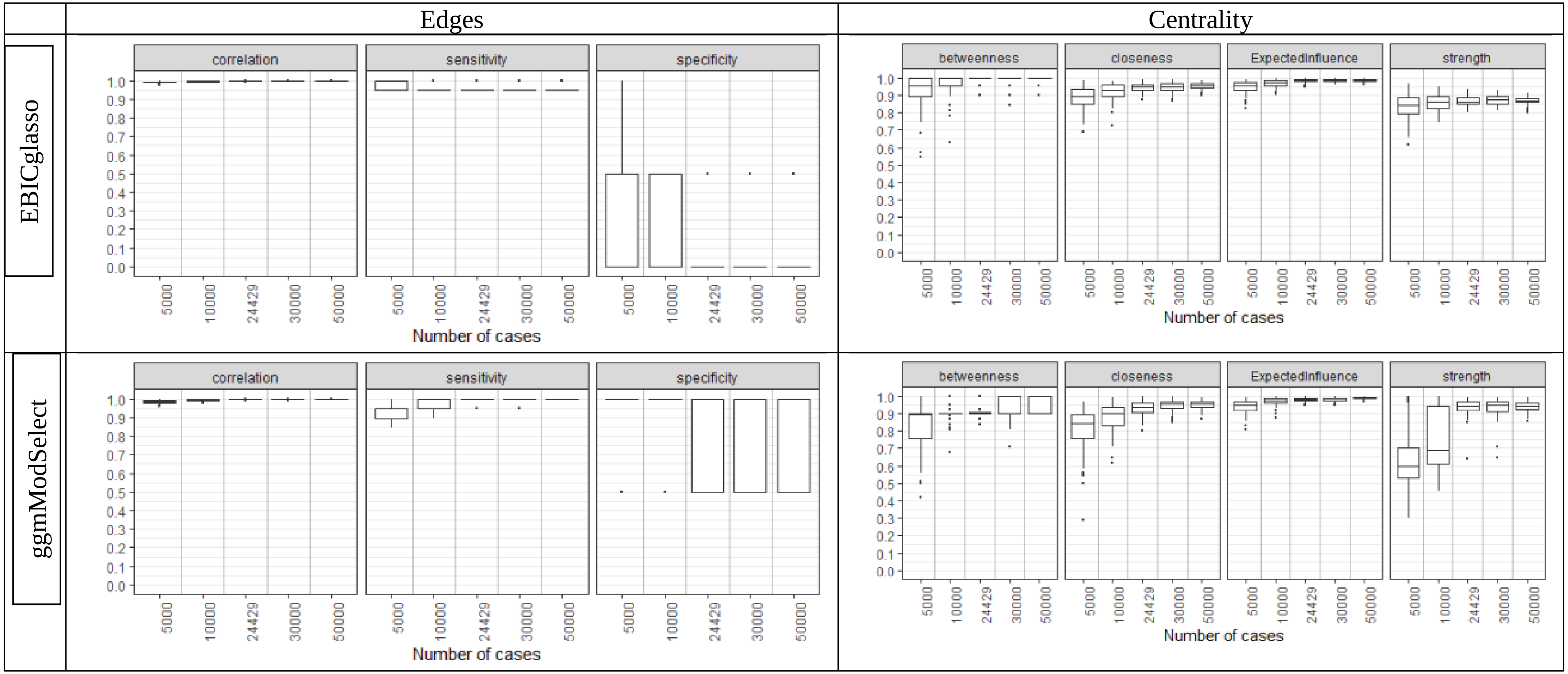

Figure S1. *P*ower analysis plots for edges and centrality measures using EBICglasso and ggmModSelect methods. Interpreted boxplots were corresponding with N = 24,590.

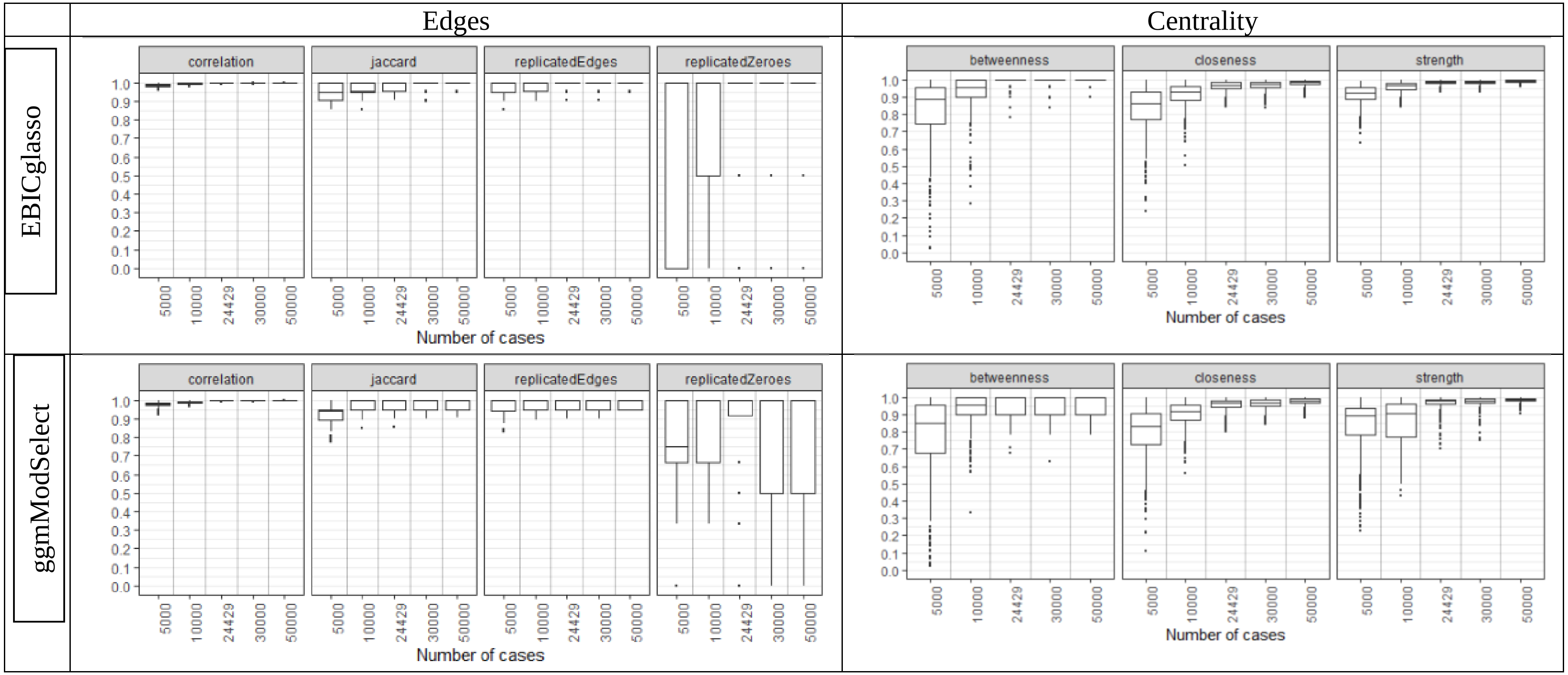

Figure S2. Replicability analysis plots for edges and centrality measures using EBICglasso and ggmModSelect methods. Interpreted boxplots were corresponding with N = 24,429.

Visual interpretations of the network

The estimated network in the manuscript is plotted using the Fruchterman-Reingold algorithm, a force-directed algorithm, aimed to produce an aesthetically pleasing network with minimal crossing edges and node overlap. This allows for an easy interpretation of network edges and groups of nodes (also known as clusters). However, distances are not interpretable with force-directed algorithms. Since we are interested in inspecting possible clusters, we followed Jones, Mair and McNally (2018) recommendations and implemented Multi-Dimensional Scaling (MDS) for plotting interpretable distances of the network. First, we checked fit for different MDS estimation methods via Shepard plots (Figure S3), and we chose the *spline* method since it provided the best fit and was more parsimonious than its nearest competitor, the *ordinal* method. The resulting network produced a plot with several node overlaps, so we plotted the same network but with minimized nodes for a better inspection of edges (Figure S2).

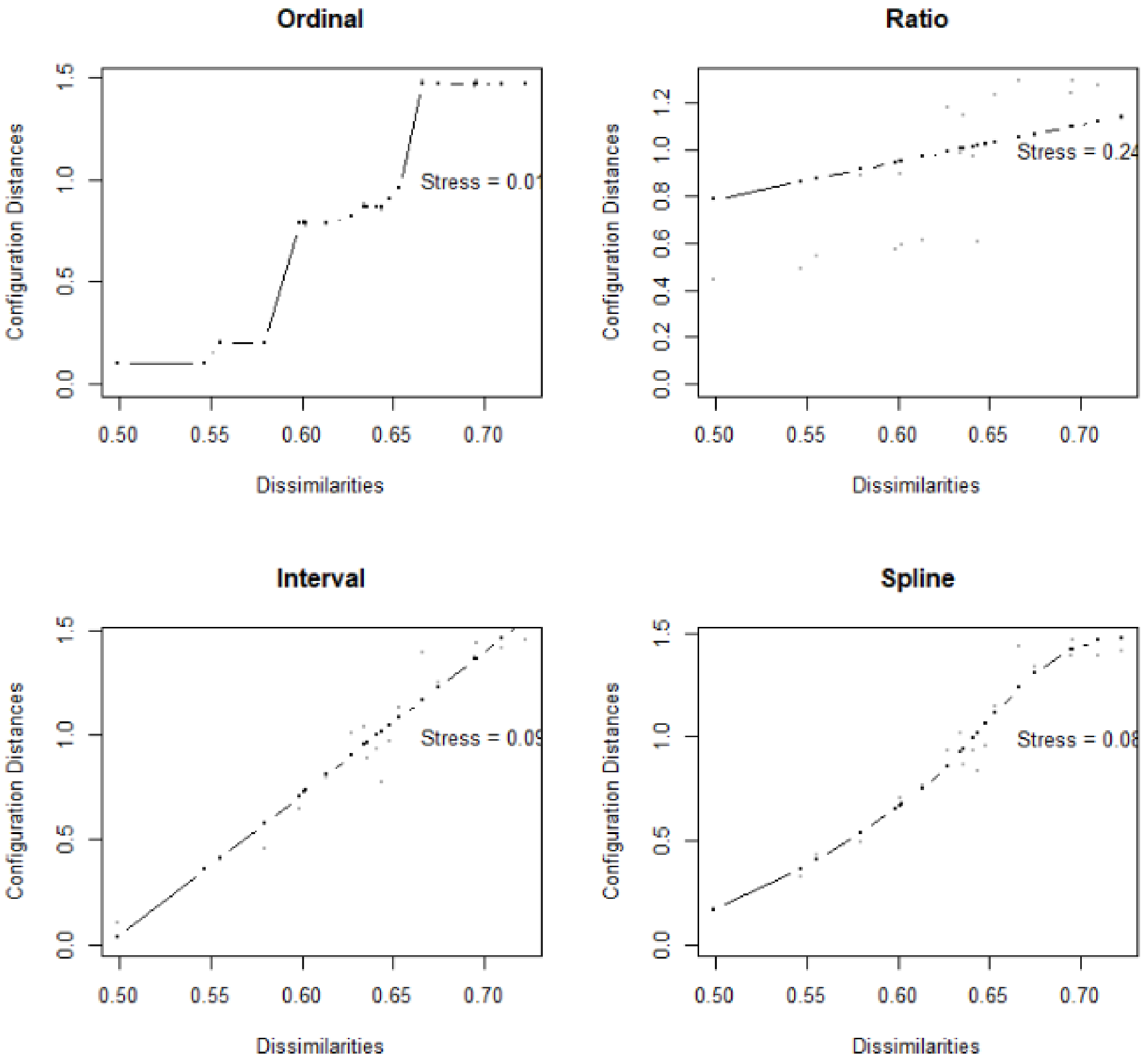

Figure S3. Shepard plots for different Multi-Dimensional Scaling methods.

The resulting plots produced a very similar spatial distribution to the Fruchterman-Reingold one. This is, a positively correlated network, with a highly-clustered "Physical" group with the items regarding racing heart, insomnia and clammy hands, and a "Psychosocial" cluster that is lightly more dispersed, with the uncomfortable, afraid, afraid to die, and anxious due to the news items. The "mspline" (i.e., spline) and "ordinal" methods produced very similar structures. Thus, we conclude that the Fruchterman-Reingold algorithm produced a sufficiently accurate representation of the network.

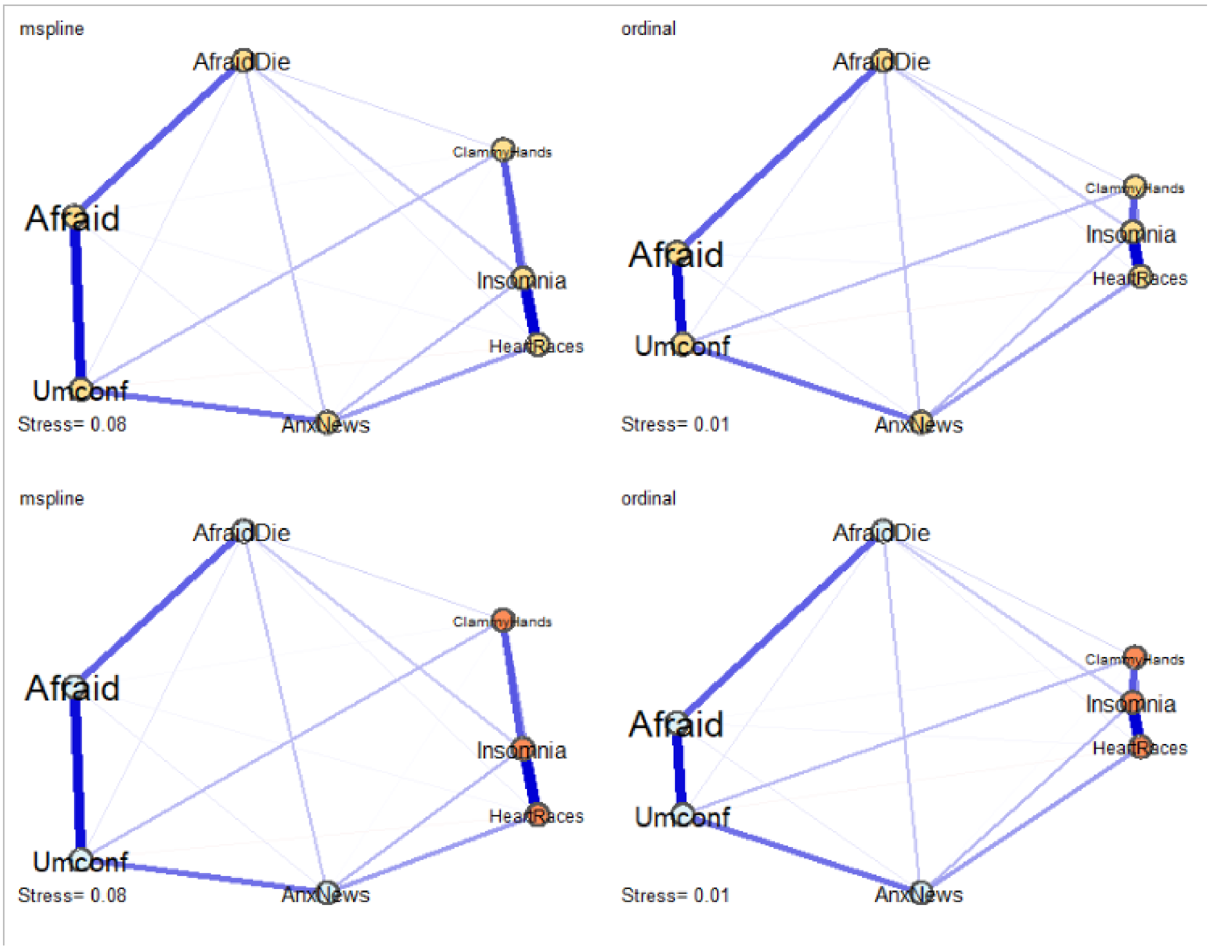

Figure S4. Multi-dimensional scaling (MDS) network for the FFMQ for the mspline and ordinal methods. Node distancing represents MDS allocations, while edges represent correlations.

Bootstrap of the estimated network

To assess network stability, we applied non-parametric and *person-drop* bootstrap (see Epskamp, Borsboom, & Fried, 2018, for details). Both bootstraps were computed with 5,000 samples. Non-parametric bootstrap was applied to assess general network stability, while *person-drop* bootstrap was applied to obtain the correlation stability (CS) indices of the network and a plot of general stability of the network. CS indices are interpreted with values of 0.25 as adequate, while values above 0.5 are ideal.

The bootstrap analyses throwed a very stable network. Figures S5 and S6 show stable solutions for the estimated network, with bootstrapped means proximate with the estimated edges (Figure S5). In addition, *person-drop* bootstrap displays very high correlations between bootstrapped and estimated edges for all re-sampling conditions. Moreover, correlation-stability coefficients show values of 0.75 for all cases of correlations in edges, thus informing of very stable correlations. Bootstrapped difference tests were not applied due to computational issues (i.e., large vectors).

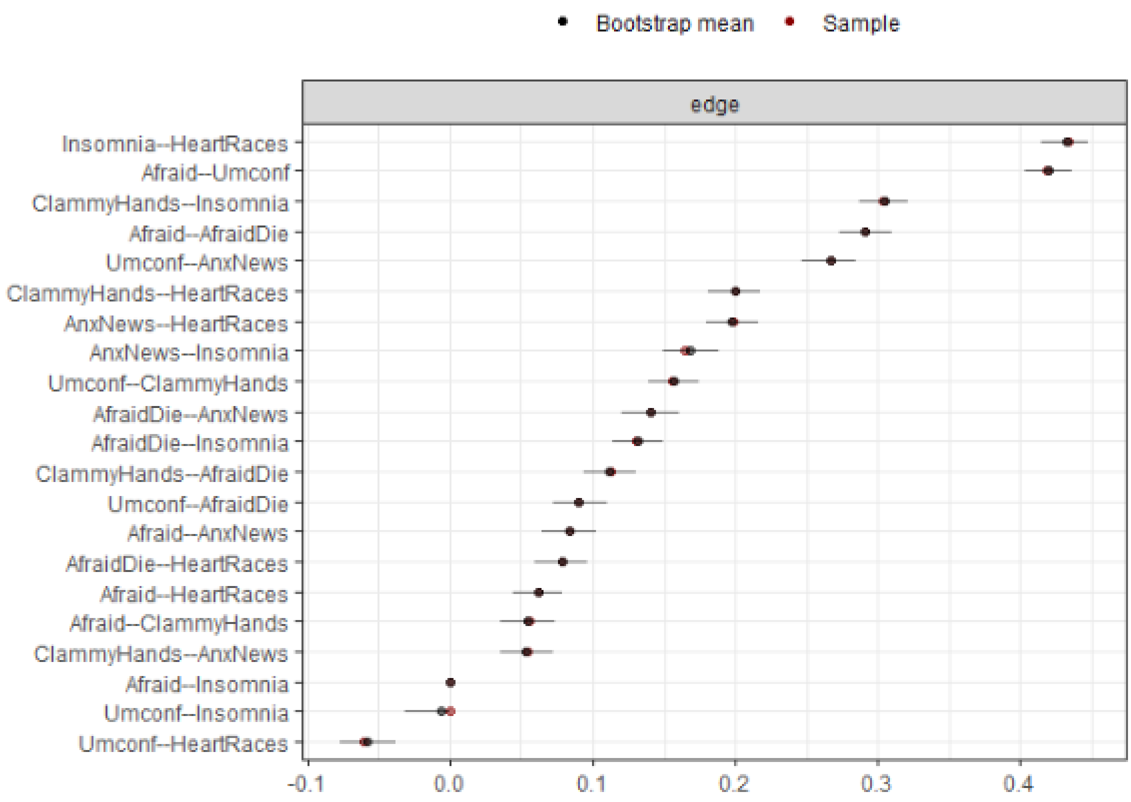

Figure S5. Bootstrapped edges on the estimated network. Horizontal axis represent edge weights, with black dots representing bootstrapped means of weights and red dots representing raw weights of the sample. Black lines represent confidence intervals.

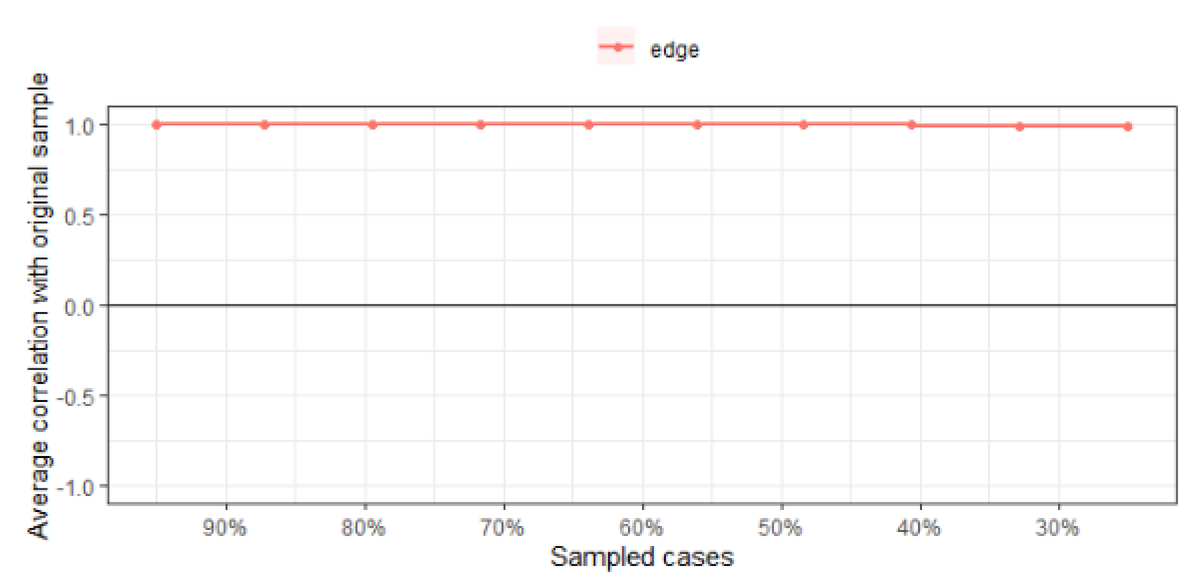
edge
Average correlation with original sample
1.0
0.5
0.0
-0.5
-1.0
90%
80%
70%
60%
50%
40%
30%
Sampled cases

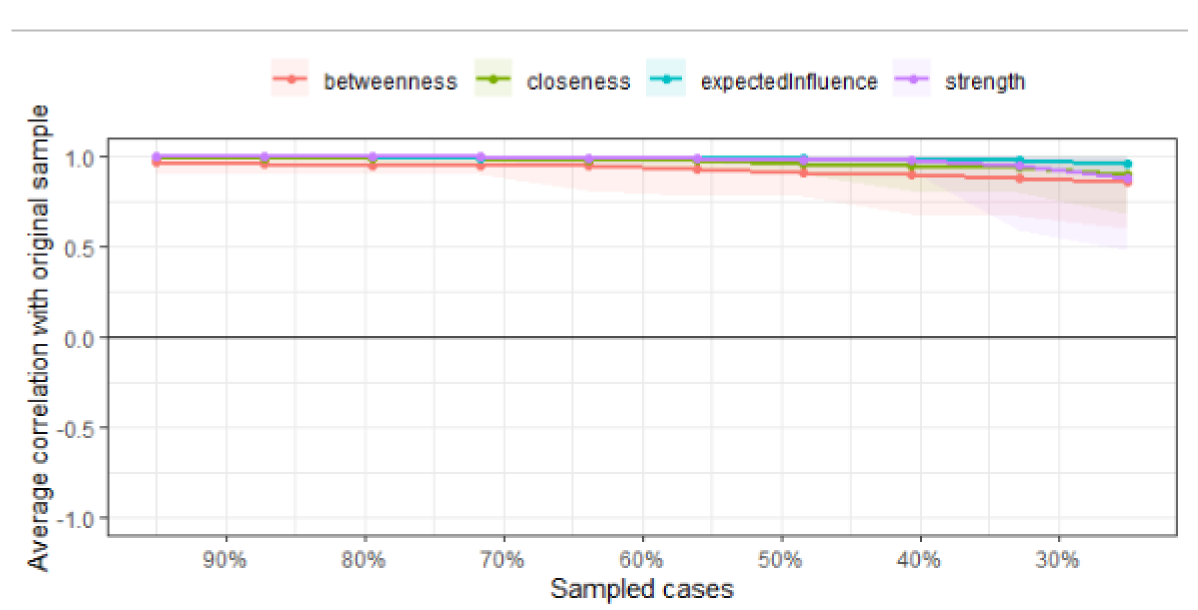
betweenness
closeness
expectedInfluence
strength
Average correlation with original sample
1.0
0.5
0.0
-0.5
-1.0
90%
80%
70%
60%
50%
40%
30%
Sampled cases

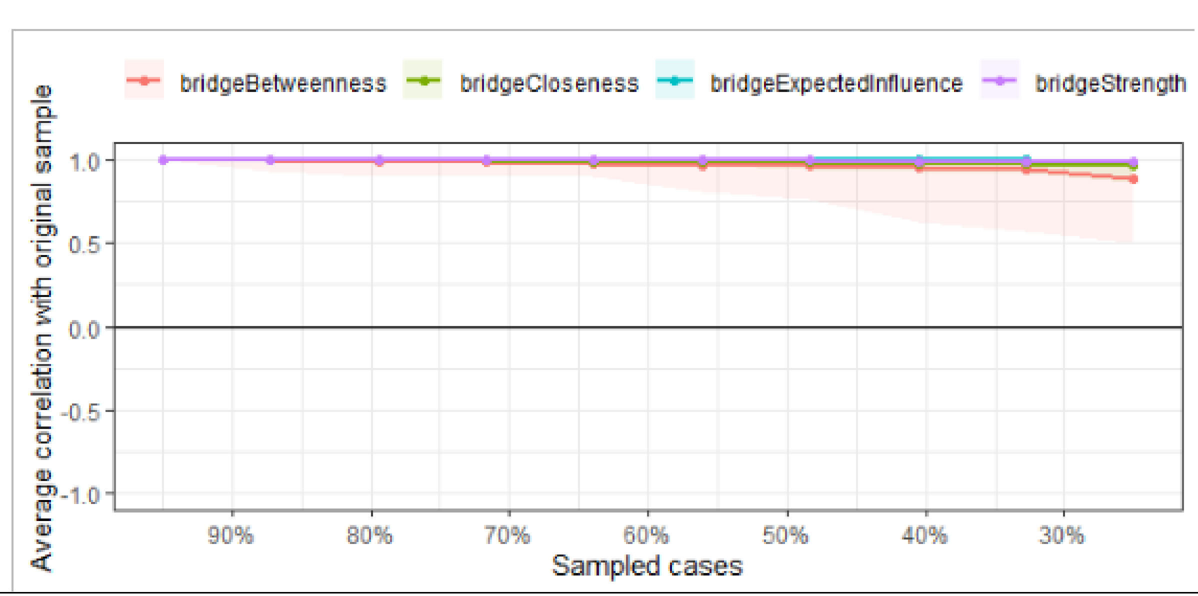
bridgeBetweenness
bridgeCloseness
bridgeExpectedInfluence
bridgeStrength
Average correlation with original sample
1.0
0.5
0.0
-0.5
-1.0
90%
80%
70%
60%
50%
40%
30%
Sampled cases

Figure S6. Edge and bidge centrality stability with 95% confidence intervals on case-drop bootstrapped network.

Bootstrap of the Exploratory Graph Analysis

We implemented two network estimation methods (gLASSO and Triangular Maximally Filtered Graph, or TMFG) crossed with *walktrap* and *Louvain* algorithms. Dimension stability was assessed with the median of proportions of proposed solutions with a confidence interval, while item stability was assessed with the median of replicated item correspondences following a certain solution. The bootstrap of the EGA showed a stable EGA solution, while primary supports the 1-factor model. The 2 and 3-factor models did not find any support. This conclusion is based on two sets of evidence. First, Table S1 displays the descriptives of bootstrapped EGA outcomes for the four bootstrap methods. All methods displayed a central estimate of 1 factor with totally relying on this solution with 100% support.

Table S1. Proposed number of dimensions for bootstrapped EGA

Type of Bootstrap	Median of dimensions	95%CI	% boots		
			1 factor	2 factor	3 factors
glasso walktrap	1	1;1	0.0	100	0.0
glasso Louvain	1	1;1	0.0	100	0.0
TMFG walktrap	1	1;1	0.0	100	0.0
TMFG Louvain	1	1;2	0.0	100	0.0

Note: Bootstraps were set for 500 boots.

Second, item stability analysis assessed if each item of the network is stable across bootstraps. As Figure S7 shows, all items display high correlations between estimated and bootstrapped samples.

Figure S7. Item stability analysis for bootstrapped EGAs estimated with EBIC-glasso or TMFG and with or without Louvain method.

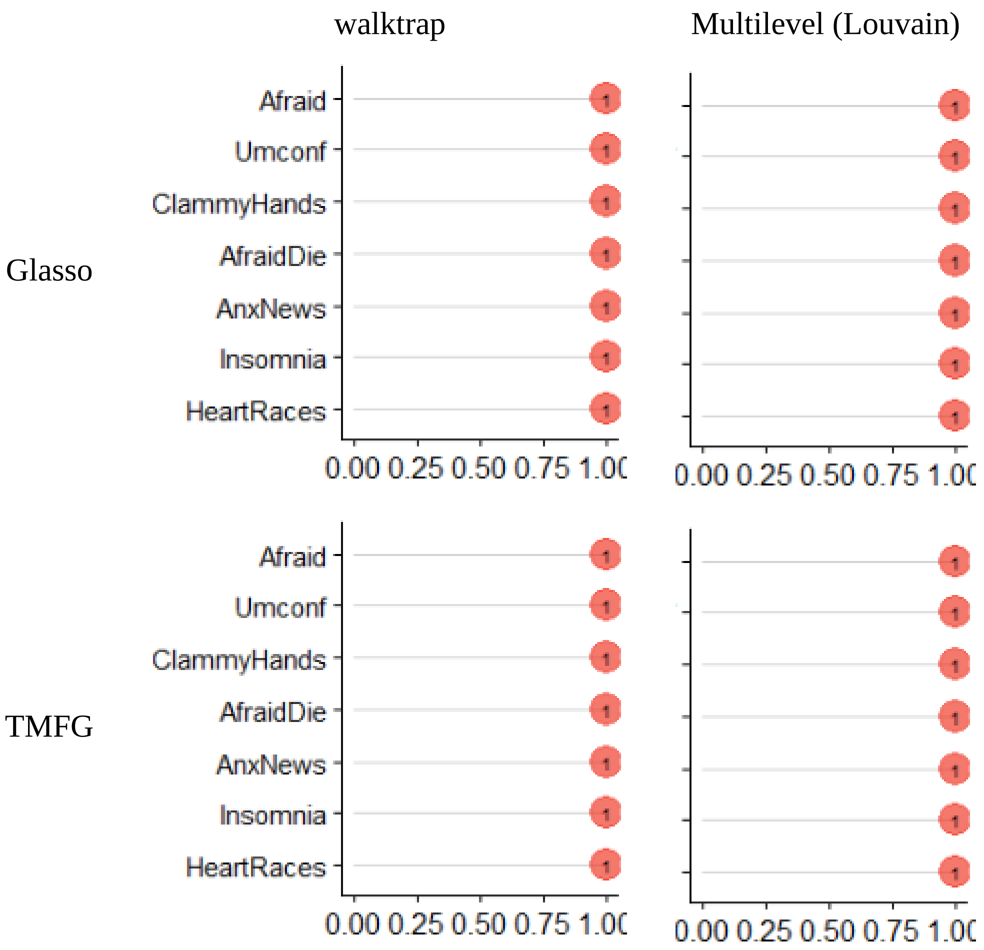

Exploratory Factor Analysis

For the sake of replicability, we implemented Exploratory Factor Analysis. We chose the polychoric correlations since all items were ordinal. To assess the number of factors, we implemented parallel analysis. We estimated factors with the Diagonal Weighted Least Squares (DWLS) with robust standard errors method, alongside the *promin* rotation method. All EFAs were bootstrapped with 500 draws and abled a 95% confidence interval for each statistic. Factor loadings were filtered and examined towards a thurstonian simple structure.

Finally, we also examined factor correlations. All these computations were performed with the FACTOR software (Ferrando & Lorenzo-Seva, 2017).

Parallel Analysis throwed strong support for the 1-factor model (Table S2). However, fit indices (CFI, TLI, RMSEA, RMSR) showed preference for the 2-factor model (Table S3). Estimating the two models resulted in factor loadings showing compatible structures in both cases (i.e., positive loadings in their corresponding factors, with small or no cross-loadings), although the 2-factor model showed more likely values. In addition, correlation between factors in the 2-factor model was positive and high (> .70).

Table S2. Parallel Analysis

Number of factors	Real-data % of variance	Mean of random % of variance	95 percentile of random % of variance
1	73.80**	29.80	40.59
2	11.13	24.11	30.65
3	5.73	18.86	23.41
4	3.62	13.97	19.06
5	3.10	8.99	14.35
6	2.59	4.25	10.24

Note. **Eigenvalues above the 95 percentile of baseline; *Eigenvalues above the mean of baseline.

Table S3. Fit indices for the 1, 2 and 3-factor models in EFA

Number of Factors	CFI	TLI	RMSEA	RMSR
1 factor	.983	.974	.102	.067
2 factor	.998	.994	.050	.018
3 factor	.999	.995	.044	.007

Therefore, we conclude that EFA provides mixed support for the 1 and 2-factor model. More concretely, the 1-factor solution was supported by Parallel Analysis. A possible explanation to this is that the correlation matrix used as input in the EFA was nor partialized nor

regularized, which could explain the support for the 1-factor model. Nevertheless, the 2-factor solution was supported by fit indices and provided a clearer loading structure.

Network comparisons

All samples showed good power and replicability values (Figures S8 – S11). The only exceptions were replicated zero correlations and betweenness, which is interpreted as the networks showing a slightly low capability of detecting null edges, while also a slightly low capability of assessing betweenness. All other parameters showed good or very good levels.

Figure S8. Power analysis for edge and centrality of males (N = 10,149) and females (N = 11,657) scores of the FC19S

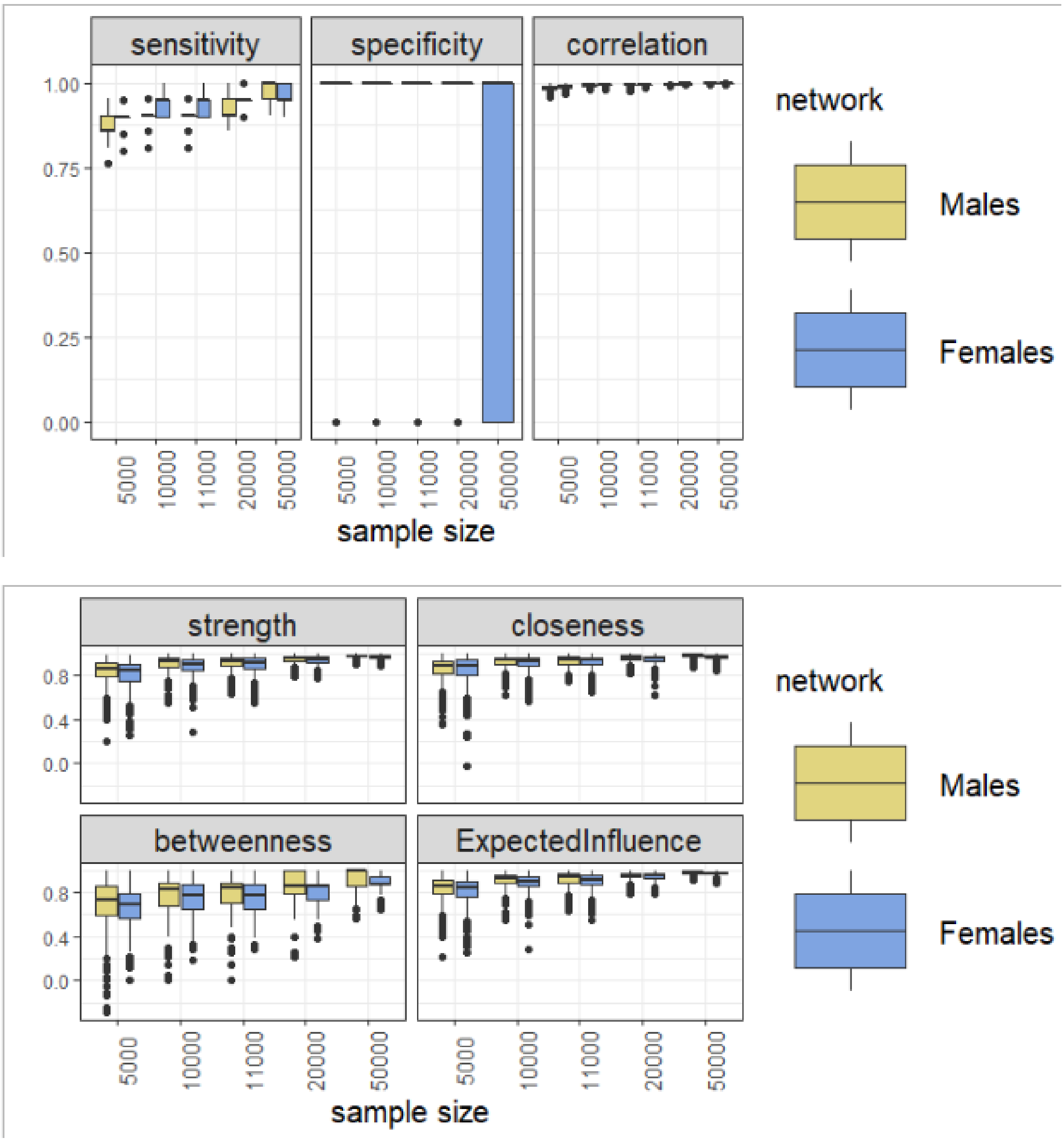

Figure S9. Replicability analysis for edge and centrality of males (N = 10,149) and females (N = 11,657) scores of the FC19S

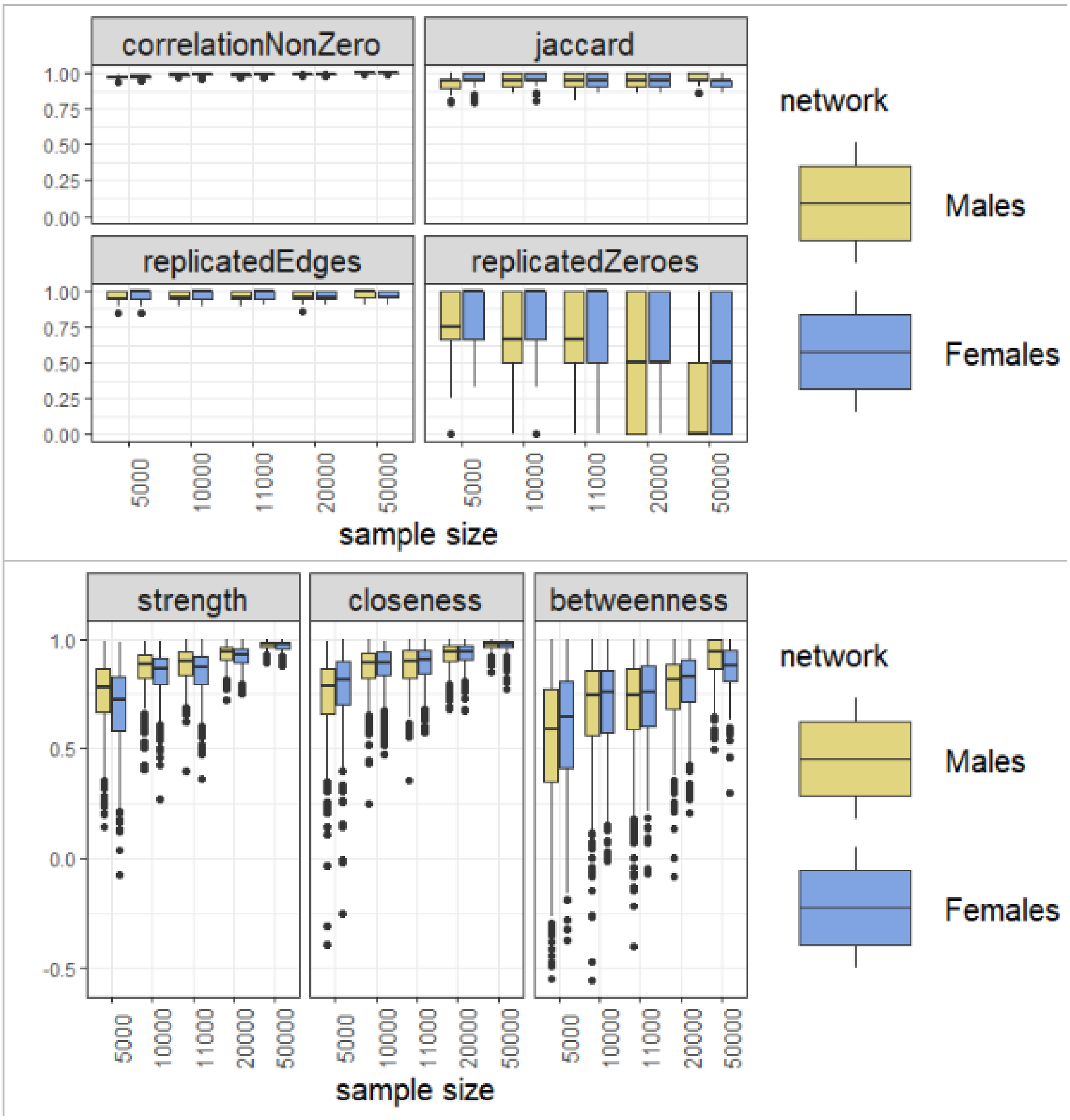

Figure S10. Power analysis for edge and centrality of Iran (N = 10,843), Bangladesh (N = 9,906) and Norway (N = 3,680) scores of the FC19S

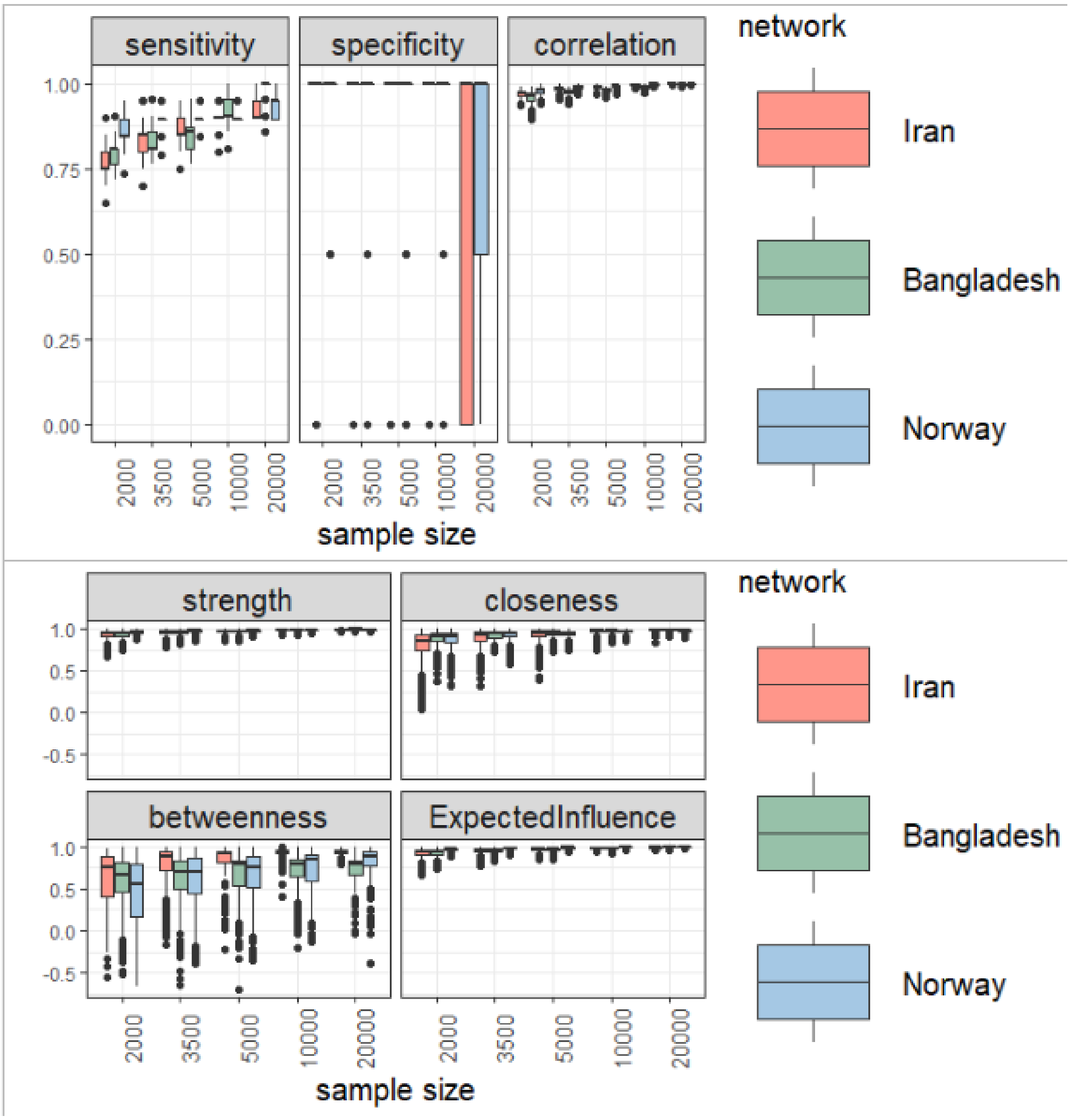

Figure S11. Replicability analysis for edge and centrality of Iran (N = 10,843), Bangladesh (N = 9,906) and Norway (N = 3,680) scores of the FC19S

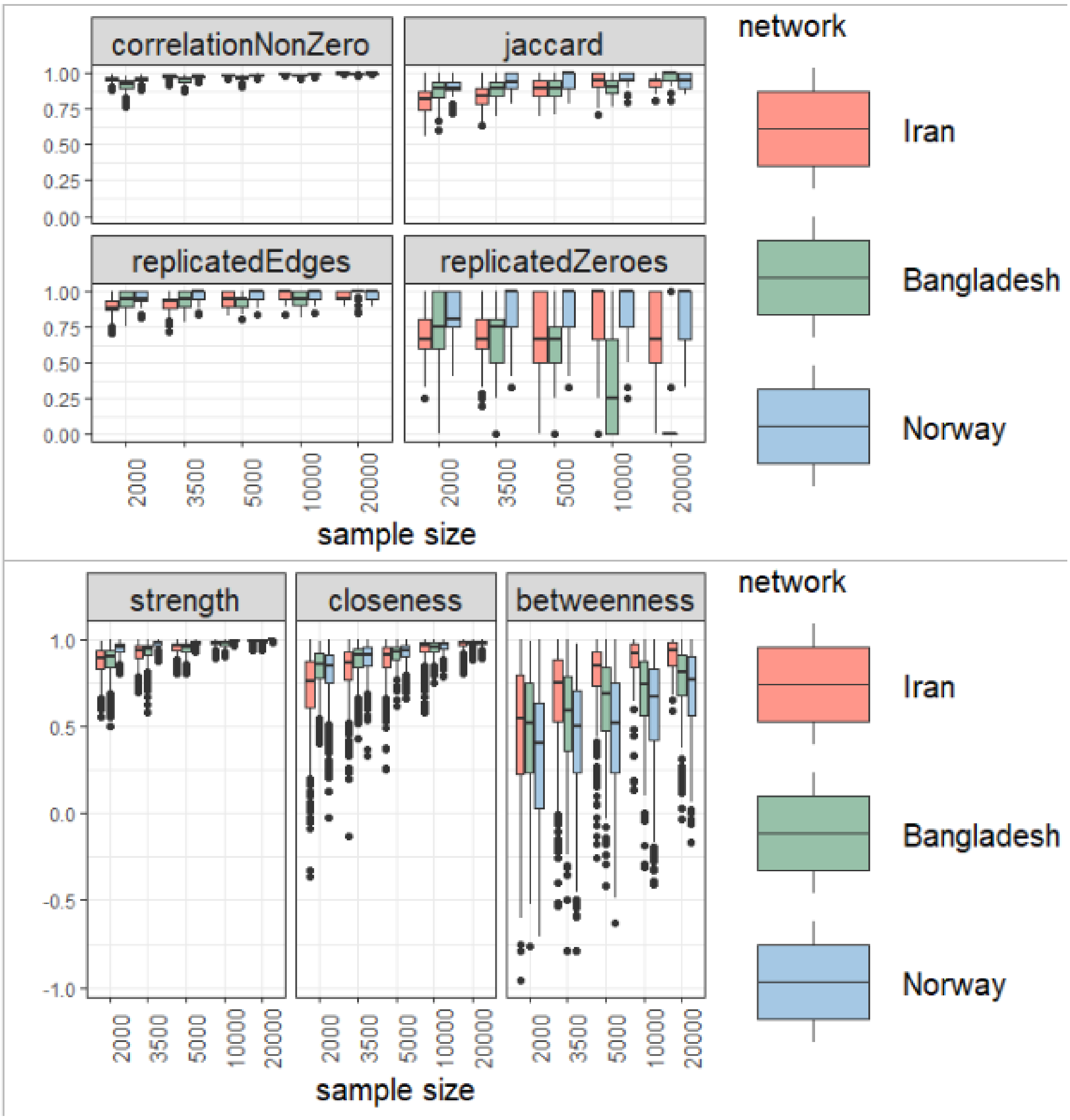
correlationNonZero
jaccard
replicatedEdges
replicatedZeroes
1.00
0.75
0.50
0.25
0.00
2000
3500
5000
10000
20000
sample size
network
Iran
Bangladesh
Norway
strength
closeness
betweenness
1.0
0.5
0.0
-0.5
-1.0
sample size

Figure S12. Power analysis for edge and centrality of young (18-29 years old, N = 13,494), mid (30-49 years old, N = 8,113) and old (>50 years old, N = 2,412) scores of the FC19S

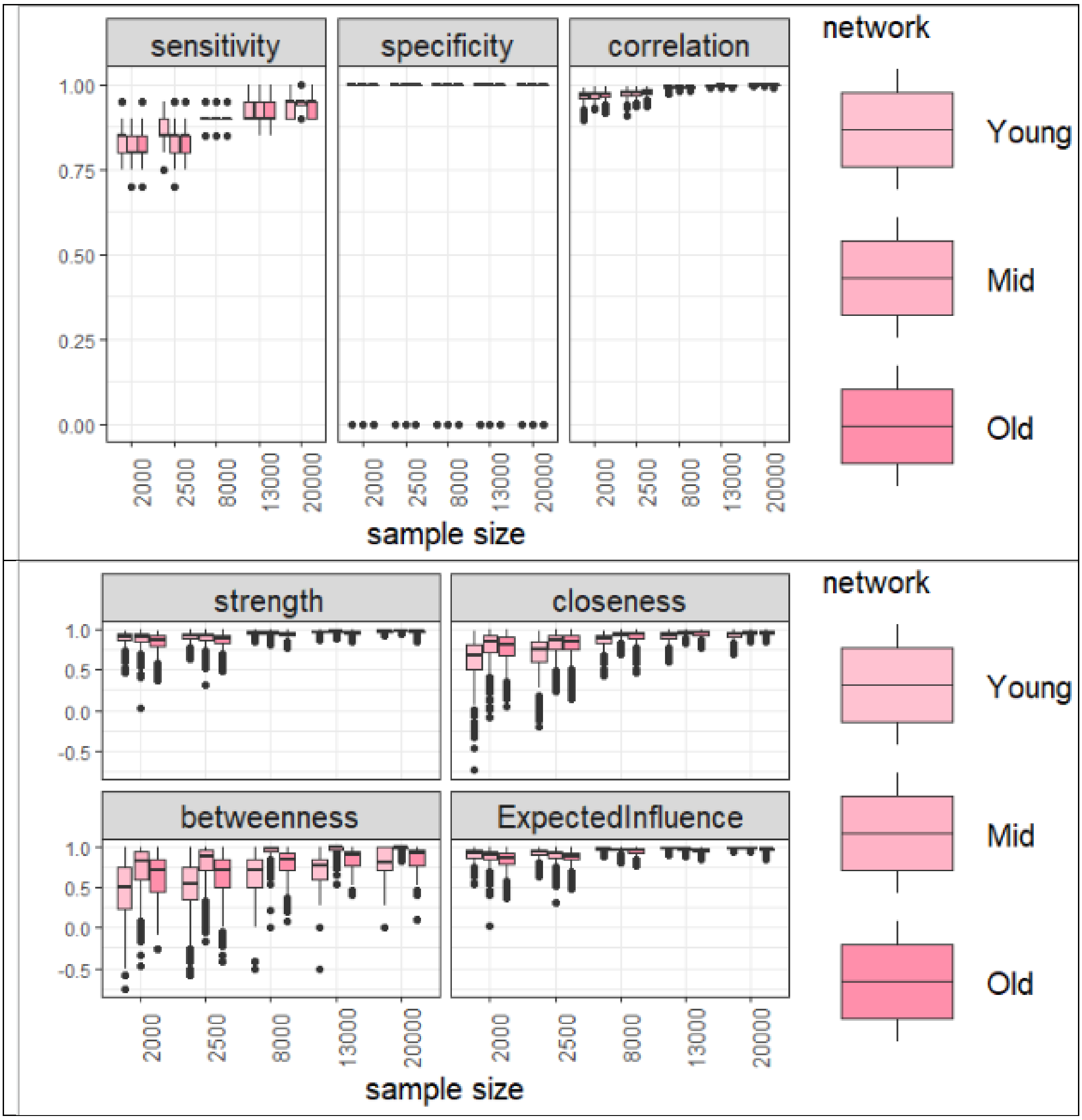

Figure S13. Replicability analysis for edge and centrality of young young (18-29 years old, N = 13,494), mid (30-49 years old, N = 8,113) and old (>50 years old, N = 2,412) scores of the FC19S

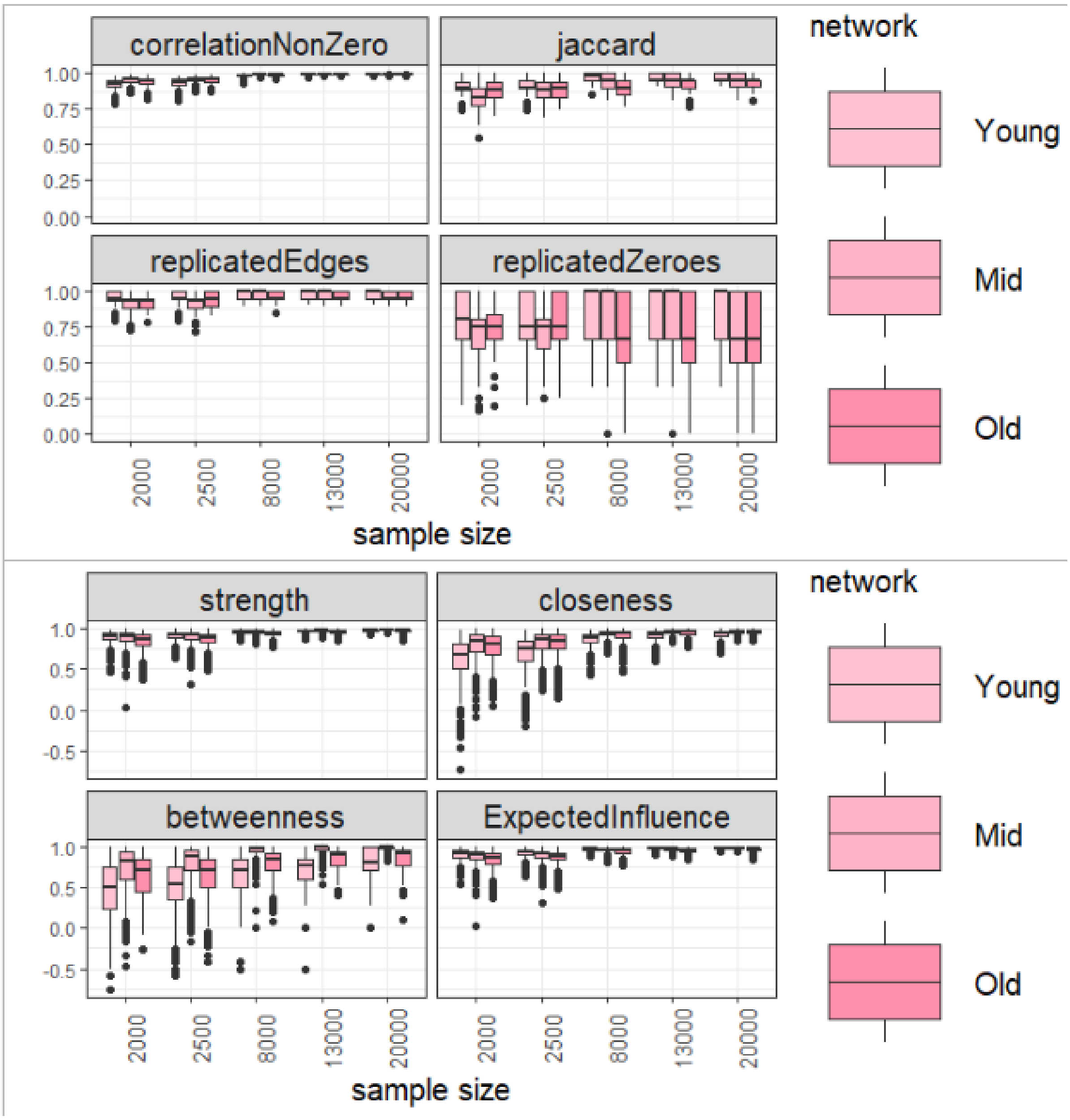

The EGAs for all subsamples showed a similar landscape than the overall network. The default EGAs supported the 2-factor solution regardless of number of steps or algorithm. However, bootstrapped EGAs provided strong support for the 1-factor solution (Table S4). Dimension stability analyses show convergent evidence with the bootstraps (Figure S14),

with 100% or >90% replicability of clusters for all subsamples. This is interpreted as no items showing noticeable instability in the EGAs. Therefore, mixed results are suggested by the EGAs, convergent with the overall network EGAs, leading to an uncertain clustering of the FC-19S between a 1 and 2-factor structures across all subsamples.

Table S4. Proposed number of dimensions for bootstrapped EGA for all subset samples

Type of Bootstrap	Median of dimensions	95%CI	% boots		
			1 factor	2 factor	3 factors
Males	1	1;1	1.0	0.0	0.0
Females	1	1;1	1.0	0.0	0.0
Iran	1	0.62;1.38	0.96	0.04	0.0
Bangladesh	1	1;1	1.0	0.0	0.0
Norway	1	1;1	1.0	0.0	0.0
Young	1	0.91;1.08	0.99	0.002	0.2
Mid	1	1;1	1.0	0.0	0.0
Old	1	0.74;1.25	0.98	0.02	0.0

Figure S14. Item stability analysis for bootstrapped EGAs estimated for males and females, Iran, Bangladesh and Norway, and young, mid and old age groups.

Males Females

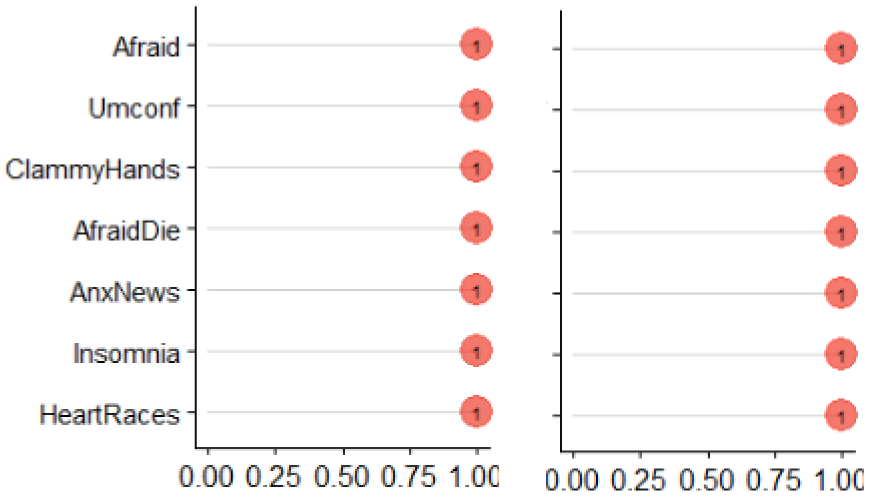

Iran Bangladesh Norway

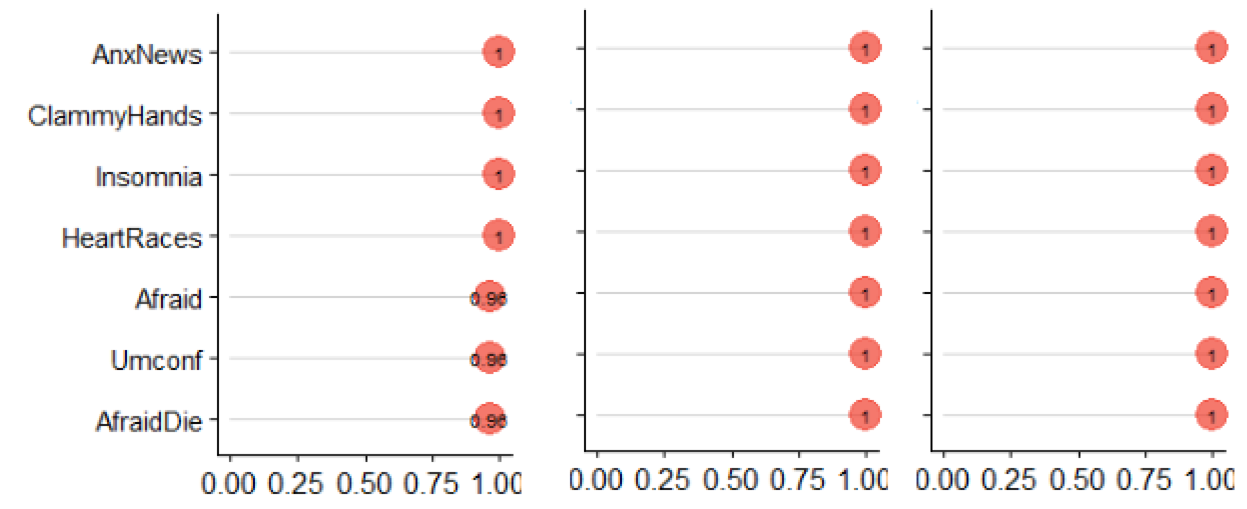

Young (18-29 years old) Mid (30-49 years old) Old (>50 years old)

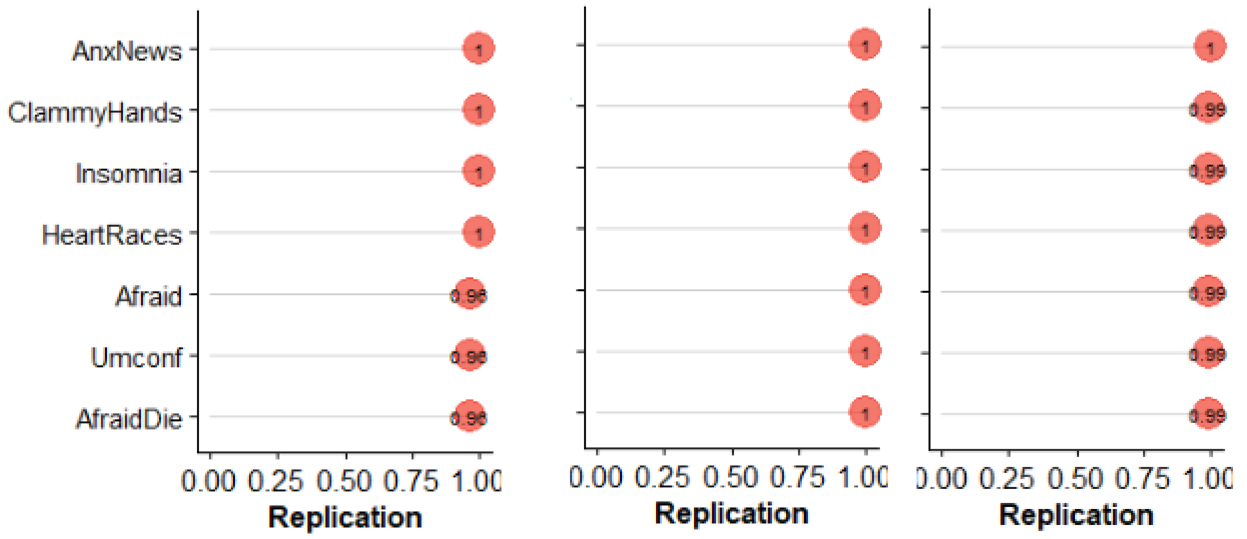

References

Epskamp, S., Borsboom, D., & Fried, E. I. (2018). Estimating psychological networks and their accuracy: A tutorial paper. *Behavior Research Methods*, 50(1), 195-212. https://doi.org/10.3758/s13428-017-0862-1

Ferrando, P.J., & Lorenzo-Seva, U. (2017). Program FACTOR at 10: origins, development and future directions. *Psicothema*, 29(2), 236-241. https://doi.org/10.7334/psicothema2016.304

Jones, P. J.; Mair, P.; and McNally, R. J. (2018) Visualizing psychological networks: A tutorial in R. *Frontiers in Psychology*, 9, 1742. https://doi.org/fpsyg.2018.01742
